# The intervention of cannabinoid receptor in chronic and acute kidney disease animal models: a systematic review and meta-analysis

**DOI:** 10.1186/s13098-024-01283-2

**Published:** 2024-02-15

**Authors:** Zihao Zhao, Qianqian Yan, Junwei Xie, Zhenjie Liu, Fengxun Liu, Yong Liu, Sijie Zhou, Shaokang Pan, Dongwei Liu, Jiayu Duan, Zhangsuo Liu

**Affiliations:** 1 Department of Integrated Traditional and Western Nephrology, the First Affiliated Hospital of Zhengzhou University, Zhengzhou, 450052 People’s Republic of China; 2 Institute of Nephrology, Zhengzhou University, Zhengzhou, 450052 People’s Republic of China; 3 Henan Province Research Center For Kidney Disease, Zhengzhou, 450052 People’s Republic of China; 4 Key Laboratory of Precision Diagnosis and Treatment for Chronic Kidney Disease in Henan Province, Zhengzhou, 450052 People’s Republic of China; 5 Academy of Medical Science, Zhengzhou University, Zhengzhou, 450052 People’s Republic of China

**Keywords:** Cannabinoid receptor, Kidney disease, Choric kidney disease, Acute kidney injury, Systematic review, Meta-analysis

## Abstract

**Aim:**

Cannabinoid receptors are components of the endocannabinoid system that affect various physiological functions. We aim to investigate the effect of cannabinoid receptor modulation on kidney disease.

**Methods:**

PubMed, Web of Science databases, and EMBASE were searched. Articles selection, data extraction and quality assessment were independently performed by two investigators. The SYRCLE’s RoB tool was used to assess the risk of study bias, and pooled SMD using a random-effect model and 95% CIs were calculated. Subgroup analyses were conducted in preselected subgroups, and publication bias was evaluated. We compared the effects of CB1 and CB2 antagonists and/or knockout and agonists and/or genetic regulation on renal function, blood glucose levels, body weight, and pathological damage-related indicators in different models of chronic and acute kidney injury.

**Results:**

The blockade or knockout of CB1 could significantly reduce blood urea nitrogen [SMD,− 1.67 (95% CI − 2.27 to − 1.07)], serum creatinine [SMD, − 1.88 (95% CI − 2.91 to − 0.85)], and albuminuria [SMD, − 1.60 (95% CI − 2.16 to − 1.04)] in renal dysfunction animals compared with the control group. The activation of CB2 group could significantly reduce serum creatinine [SMD, − 0.97 (95% CI − 1.83 to − 0.11)] and albuminuria [SMD, − 2.43 (95% CI − 4.63 to − 0.23)] in renal dysfunction animals compared with the control group.

**Conclusions:**

The results suggest that targeting cannabinoid receptors, particularly CB1 antagonists and CB2 agonists, can improve kidney function and reduce inflammatory responses, exerting a renal protective effect and maintaining therapeutic potential in various types of kidney disease.

**Supplementary Information:**

The online version contains supplementary material available at 10.1186/s13098-024-01283-2.

## Introduction

Kidney diseases including chronic kidney disease (CKD) and acute kidney injury (AKI) [1, 2], are a significant global health problem, affecting millions of people worldwide [3, 4]. CKD is a common endpoint disease of kidney disease of multiple etiologies, including diabetic nephropathy, obesity-related nephropathy, chronic tubulointerstitial injury, and recurrent AKI [5–7]. Renal fibrosis and mild inflammation are the basis of CKD progressing to end-stage renal disease [8, 9]. Despite advances in treatment, the incidence and prevalence of kidney disease continue to rise, and new therapies are urgently needed to prevent or slow disease progression [10]. Recent studies have suggested that targeting the endocannabinoid system, particularly the cannabinoid receptor 1 (CB1) and cannabinoid receptor 2 (CB2), may have therapeutic potential in various kidney diseases [11, 12]. While a growing body of evidence suggests that cannabinoid receptors play a role in the regulation of renal function and in the pathogenesis of kidney diseases, the intervention of cannabinoid receptors in renal diseases is a relatively uncharted territory, with potential implications for both therapeutic and adverse effects. A comprehensive understanding of these interactions, specifically in the setting of kidney disease models, is critical to harnessing their potential therapeutic benefits while mitigating risks.

Meta-analysis is a powerful tool for synthesizing and analyzing data from multiple studies, providing a comprehensive overview of the available evidence and increasing statistical power, even in animal study [13, 14]. In the present study, we aim to perform a systematic review and meta-analysis of preclinical animal studies investigating the effects of cannabinoid receptor modulation on kidney disease. Specifically, we will analyze the effects of cannabinoid receptor agonists and antagonists on kidney function, histological changes, inflammation, and other relevant outcomes in animal models of kidney disease. By synthesizing and analyzing data from multiple studies, we hope to provide a more comprehensive understanding of the role of cannabinoid receptors in kidney disease, and to identify potential therapeutic targets for future studies in this field.

## Materials and methods

### Search strategy

PubMed, Web of Science databases, and EMBASE were searched for publications using the following search terms: “kidney disease”, “renal function”, “cannabinoid receptor”, “cannabinoids” and “animal”, with the last search performed on 20 August 2021. Detailed search strategies are shown in the Additional file 1. No language restriction filter was applied. Furthermore, we screened the reference lists of the papers identified through database search for other potentially appropriate studies. When necessary, we asked the contact author of individual studies for more information by email.

### Selection criteria

All articles acquired by the search were reviewed, and irrelevant publications were removed by scanning the title and abstract. The selected articles were further assessed by full-text reading. Two investigators independently (Z. Z. and J.D.) achieved the study selection, with any differences determined by common discussion and judgment of a third reviewer (D.L.), if consensus was not reached. Studies that conformed to the following criteria were considered possibly qualified: (1) Animal models of acute and chronic kidney disease; (2) modulators of cannabinoid receptors (including agonists and antagonists) or genetic modifications of CB1 or CB2 were used as interventions, with corresponding control groups set up; (3) The primary outcomes were albuminuria, blood urea nitrogen (BUN), serum creatinine (Scr), kidney weight/body weight ratio (KW/BW) and pathology of renal tissue, and (4) the secondary outcomes were blood glucose, body weight (BW), mechanistic pathway indicators of cannabinoid receptor system involved in kidney tissue injury. Human studies, in vertebrate animal and in vitro, ex vivo experimental studies will be excluded. The protocol of this meta-analysis was registered in the International Prospective Register of Systematic Reviews (PROSPERO) (CRD42021272950).

### Quality assessment

Data extraction and quality assessment were independently performed by two investigators (Z.Z. and D.L.). All eligible study reports were used for the quality assessment using the Systematic Review Center for Laboratory Animal Experimentation (SYRCLE)’s risk of bias tool for animal studies [15] and for data collection. Any discrepancies were resolved through discussion to reach a consensus. Results are visualized using the “robvis” package in R.

### Data extraction

Study quality, study characteristics such as first author, year of publication, study design and sample size, animal characteristics such as species and sex, disease model and methods of establishment, target receptor, intervention protocol including dose administration and duration, and primary and secondary outcomes index were collected into standardized extraction forms. In the case of primary and secondary outcomes, when there were multiple measurements taken at various intervals (e.g., bi-weekly readings of blood glucose, weight, urine protein, etc.), we solely considered the data from the final time point, which corresponds to the post-intervention period. When interventions with different dose subgroups existed, we extracted each results and combined them into one treatment group using a formula provided by Cochrane [16] via the web tool StatsToDo (https://www.statstodo.com/). Using WebPlotDigitizer (https://automeris.io/WebPlotDigitizer) to extract data displayed only as graph curves. For the meta-analysis, studies were required to report the number of animals per group, the mean, and a measure of variance. Standard error of mean (SEM) was converted to standard deviation (SD) value according to the method recommended by Cochrane Handbook [16], i.e., SD = SE × $\sqrt{n}$.

### Statistical analysis

This meta-analysis was performed using R 4.1.2 software (https://www.r-project.org). The standardized mean difference (SMD) have been used to assess the effects of treatment among the different continuous scales of measurement. The random-effects model was used to calculate the pooled effect by using “meta” and “metafor” package. The *I*^2^ and *Q* test was used to quantify the degree of heterogeneity among studies [17]. *I*^2^ > 50% considered to reveal higher heterogeneity among the included studies.

### Subgroup and sensitivity analyses

Subgroup analyses were conducted stratified by the species, the intervention is pharmacological or genetic, year of study published, disease models for chronic or acute, and method of model establishment. Sensitivity analysis was used to explore the impact of a single study on the overall risk estimate, and was carried out by sequentially omitting one study in each iteration with the “metainf” package in R software.

### Publication bias

Publication bias was examined by funnel plot and the Egger's test [18]. To evaluate the possibility of publication bias, we visually examined the funnel plots for asymmetry. Additionally, we utilized trim and fill analysis to address any observed funnel plot asymmetry. This involved estimating and including the potentially missing studies on the left-hand side of the plot, which allowed us to recalculate the overall effect size.

## Results

### Characteristics and quality of the retrieved studies

**Fig. 1:**
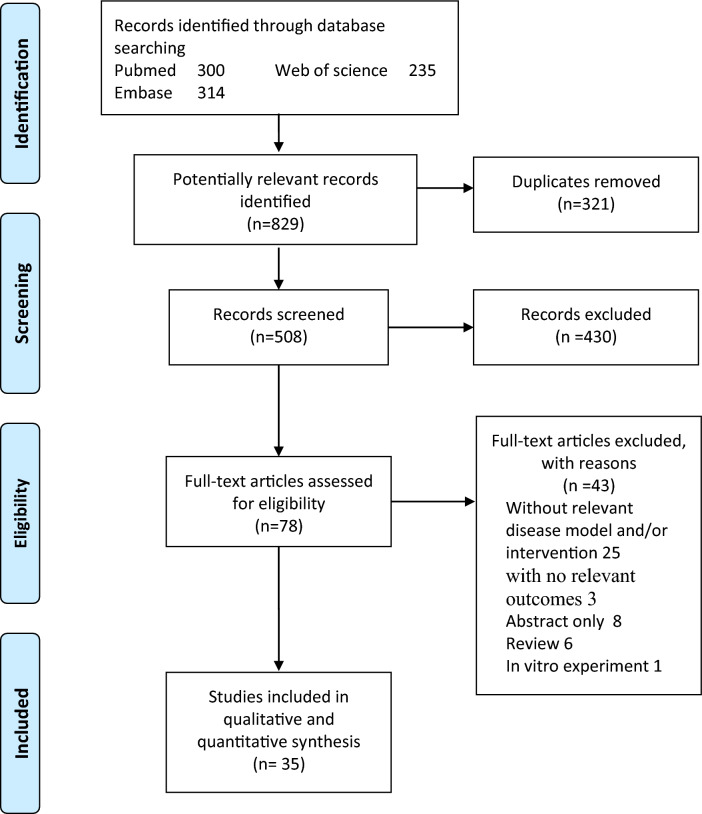
A flow diagram of the literature screening and selection. Reported in accordance with the PRISMA guidelines

We identified 829 potentially relevant references from database searches (Fig. 1). Then, 321 duplicate studies were removed leaving a total of 508 potentially relevant citations identified from the initial stage of the literature search. Excluded 430 studies after screening the titles and abstracts and read the full text of the remaining 78 studies. We subsequently excluded 6 reviews, 8 abstracts only, 25 studies that contained unrelated disease models and/or interventions, 3 studies with no relevant outcomes and one study only contained in vitro experiment. Finally, a total of 35 studies were included in the analysis [19–53]. The detailed characteristics of the included studies are shown in Table 1 and Additional file 1: Table S1.

**Table 1:** The characteristics of the included studies

	Study	YEAR	Country	Species	Sex	Animal disease model	Target receptor
1	Janiak P	2007	France	Rat	Male	Obese fa/fa Zucker rats	CB1R antagonist
2	Federica B	2010	Italy	Mouse	Male	STZ-induced diabete C57BL7/J mice	CB1R antagonist
3	Partha M	2010	USA	Mouse	Male	Cisplatin-induced renal dysfunction C57BL/7 J mice	CB1R antagonist, genetic deletion of CB1R
4	Partha M2	2010	USA	Mouse	Male	Cisplatin-induced renal dysfunction C57BL/7 J mice	CB2R knockout, CB2R agonist
5	James C	2010	Canada	Rat	Male	JCR:LA-cp obese cp/cp rats	CB1R antagonist
6	Federica B	2011	Italy	Mouse	Male	STZ-induced diabete C57BL7/J mice	CB2R agonist
7	Mónica A	2012	Spain	Rat	Male	Diet-induced obesity Wistar rats	CB1R antagonist
8	Béla H	2012	USA	Mouse	Male	Cisplatin-induced renal dysfunction C57BL/7 J mice	CB2R knockout, CB2R agonist
9	D. H. Nam	2012	Korea	Mouse	Male	db/db diabetic mice	CB1R antagonist
10	Tang Y	2012	USA	Mouse	Male	Diet-induced obese AKR/J Mice	CB1R-ASO
11	Federica B	2014	Italy	Mouse	Male	STZ-induced diabete C57BL7/J mice	CB2R knockout
12	Jourdan T	2014	USA	Rat	Male	Zucker Diabetic Fatty rats	Peripheral CB1R antagonist
13	Chun-L L	2014	China (Taiwan)	Rat and Mouse	Male	STZ-induced diabete rats and FVB/N mice	CB1R transgenic,CB1R antagonist and CB1R ASO
14	Yung-C H	2015	China (Taiwan)	Rat and Mouse	Male	Wistar rats	CB1R transgenic, CB1R agonist
15	Kayte A	2015	Australia	Rat	Male	Diet-induced obesity Sprague–Dawley rats	CB1R antagonist
16	Lola L	2015	France	Mouse	Unclear	UUO renal fibrosis	CB1R antagonist
17	Chih-Y L	2015	China (Taiwan)	Mouse	Male	Partial nephrectomy uremia	CB1R antagonist
18	Kayte A	2016	Australia	Rat	Male	Diet-induced obesity Sprague–Dawley rats	CB2R agonist
19	Partha M	2016	USA	Mouse	Male	Cisplatin-induced renal dysfunction C57BL/7 J mice	CB2R agonist, CB2R knockout
20	Carlamaria Z	2016	Italy	Mouse	Male	BTBR ob/ob mice	CB2R agonist
21	Jourdan T	2017	USA	Rat	Male	Zucker diabetic fatty rats	Global deletion of CB1R
22	Shiran U	2017	Israel	Mouse	Male	High-fat diet mice	Renal proximal tubular cells-specific CB1R-null
23	F Barutta	2018	Italy	Mouse	Male	STZ-induced diabete C57BL7/J mice	Peripheral CB1R antagonist
24	Liad H	2018	Israel	Mouse	Male	STZ and Akita mice	Peripheral CB1R antagonist
25	Jourdan T	2018	USA	Mouse	Male	STZ-induced diabete C57BL7/J mice	Podocyte-specific Cnr1 deletion
26	Jeffrey D	2018	India	Mouse	Male	Ischemia–reperfusion injury C57BL/7 J mice	CB2R agonist
27	Li Z	2018	China	Mouse	Male	UUO and UIRI male BALB/c mice	CB2R inverse agonist
28	Murat Ç	2019	Turkey	Rat	Male	Renal ischemia reperfusion Sprague–Dawley rats	CB2R agonist
29	Eszter T	2020	USA	Mouse	Male	C57BL/6 J male mice of HRS, induced by BDL	CB2R agonist
30	Shiran U	2020	Israel	Mouse	Male	High-fat diet mice	Peripheral CB1R antagonist
31	Isabel G	2021	Spain	Mouse	Male	STZ-induced diabete C57BL7/J mice	Dual CB1R antagonist/CB2R agonist CBD
32	Jayarami R	2021	India	Rat	Unclear	STZ-induced diabete rats	Peripheral CB1R antagonist
33	Li Z	2021	China	Rat	Male	Chronic intermittent hypoxia Sprague–Dawley rat	CB1R antagonist
34	Shan Z	2021	China	Mouse	Male	Unilateral nephrectomy and D-gal aged C57BL/7 mice	CB2R knockout
35	Shan Z2	2021	China	Mouse	Male	UIRI and UUO C57BL/7 mice	CB2R agonist

**Fig. 2:**
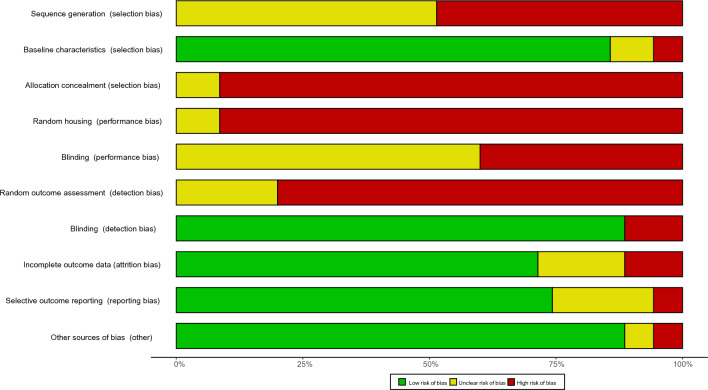
Summary of risk of bias assessments for included studies by using the Systematic Review Center for Laboratory Animal Experimentation (SYRCLE)’s risk of bias tool: percentages of judgments for each bias item

Figure 1 All studies were published between 2007 and 2021. Of the studies included, 33 rodent models were male [19–33, 35–49, 51–53], and 2 did not mention sex [34, 50]. Ten of the animal models studied were rats [19, 23, 25, 30, 33, 36, 39, 46, 50, 51], 23 were mice [20–22, 24, 26–29, 34, 35, 37, 38, 40–45, 47–49, 52, 53], and 2 included both rat and mouse models [31, 32]. Disease models include chronic kidney injury models that include streptozotocin (STZ) or genetically induced diabetes [20, 24, 27, 29–31, 39, 41–43, 49, 50], diet or genetically induced obesity [19, 23, 25, 28, 32, 35, 38, 40, 48], chronic intermittent hypoxia [51], bile duct ligation induced hepatorenal syndrome [47] and acute kidney injury models that include unilateral ureteral obstruction, ischemia–reperfusion, partial nephrectomy, and cisplatin induction [21, 22, 26, 34, 35, 37, 44–46, 52, 53]. The target receptors for the intervention included CB1 antagonist and knockout [19–21, 23, 25, 27, 28, 30, 31, 33–35, 39–43, 48–51], CB2 agonist [22, 24, 26, 36–38, 44, 46, 47, 53], CB2 antagonist and knockout [22, 26, 29, 37, 45, 52], and CB1 agonist [32] (the summary as shown in Table 2). The study quality was assessed according to the SYRCLE. Eighteen studies reported random assignment of animals, but no study described a random component in the specific sequence generation process. No studies have specifically reported the method of allocation concealment and randomly placed animals in animal housing. Baseline characteristics (selection bias), blinding (detection bias), incomplete outcome data (attrition bias), selective outcome reporting (reporting bias) and other sources of bias were mostly well performed. The risk-of-bias assessment of the included trials was summarized in Fig. 2 and Additional file 1: Fig. S1.

**Table 2:** Summary of Small Molecule Drugs Involved in Included Studies

Target	Drug	Synonyms	CAS No	IC 50	Formula
CB1R antagonist	SR141716	Rimonabant	168,273-06-1	1.8 nM	C_22_H_21_Cl_3_N_4_O
	AM251	–	183,232-66-8	8 nM	C_22_H_21_Cl_2_IN_4_O
	AM281	–	202,463-68-1	9.91 nM	C_21_H_19_Cl_2_IN_4_O_2_
	LH-21	–	611,207-11-5	–	C_20_H_20_Cl_3_N_3_
	JD5037	–	1,392,116-14-1	1.5 nM	C_27_H_27_Cl_2_N_5_O_3_S
	AM6545	–	1,245,626-05-4	1.7 nM	C_26_H_23_Cl_2_N_5_O_3_S
	SLV319	Bipinabant	464,213-10-3	7.8 nM(Ki)	–
	( +)-CBD-HPE	–	–	3.1 nM(Ki)	-
CB2R agonist	HU-308	–	256,934-39-1	22.7 nM(Ki)	C27H42O3
	AM1241	–	444,912-48-5	3.4 nM(Ki)	C_22_H_22_IN_3_O_3_
	β-Caryophyllene	(–)-trans-Caryophyllene	87-44-5	–	C_15_H_24_
	LEI-101	–	1,228,660-00-1	7.5 nM(Ki)	C23H25FN4O4S
	HU-910	–	–	6 nM(Ki)	/
	SMM-295	–	1,054,451–22-7	–	C20H20O2S
	JWH-133	–	259,869–55-1	–	C22H32O
CB2R antagonist	AM630	6-Iodopravadoline	164,178–33-0	31.2 nM	C23H25IN2O3
	XL-001	–	–	0.5 nM(Ki)	–

### Effect of CB1 antagonist and knockout on renal function

#### Primary outcomes

##### BUN

**Fig. 3:**
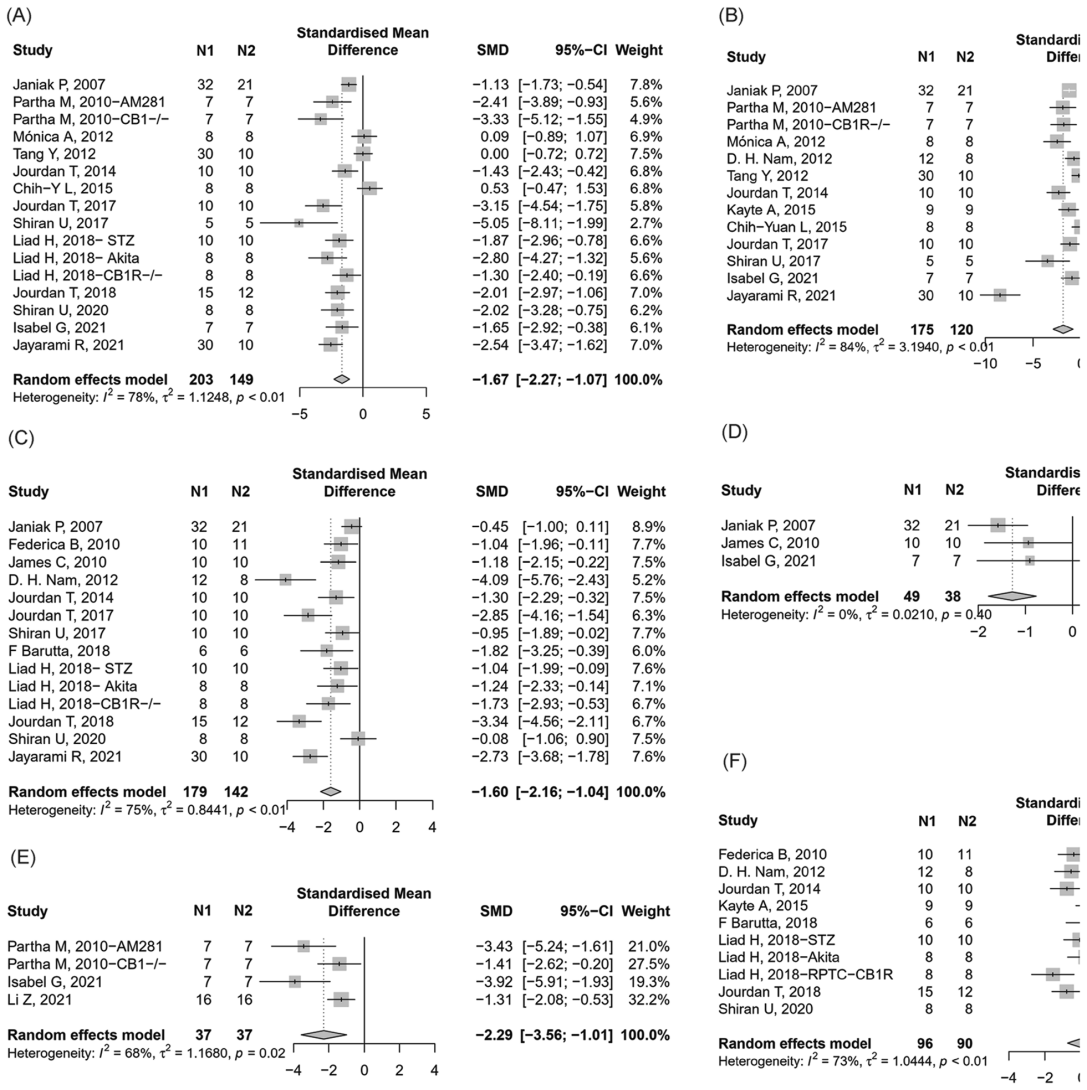
Meta-analysis and forest plot of effects of CB1 antagonist and knockout on primary outcomes including blood urea nitrogen (**A**), serum creatinine (**B**), albuminuria (**C**), glomerular damage score (**D**), tubular damage score (**E**), and kidney weight/body weight ratio (**F**). N1 denotes the number of animals in the treatment group and N2 denotes the number of animals in the control group. The size of the square indicates the weight (%) of each study, the horizontal line shows the 95% confidence interval (95%-CI) of the individual standardized mean difference (SMD), and the black diamond represents the combined SMD and 95%-CI

Thirteen studies [19, 21, 25, 28, 30, 35, 39, 40, 42, 43, 48–50]reported BUN found that the blockade or knockout of CB1 group could significantly reduce BUN in renal dysfunction animals compared with the control group (Fig. 3A, 16 items, n = 352; SMD, − 1.67; 95% confidence interval (CI), − 2.27 to − 1.07; *P* < 0.0001; *I*^2^ = 78%).

##### Scr

A total of 12 included studies reported Scr after intervention with CB1 knockout or drug blockade [19, 21, 25, 27, 28, 30, 33, 35, 39, 40, 49, 50]. In the random-effect model, the blockade or knockout of CB1 had a significantly effect on Scr (Fig. 3B, 13 items, n = 295; SMD, − 1.88; 95% CI − 2.91 to − 0.85; *P* < 0.0001; *I*^2^ = 84%) compared to control.

##### Albuminuria

A total of 12 included studies [19, 20, 23, 27, 30, 39–43, 48, 50] reported albuminuria after intervention with CB1 knockout or drug blockade, of which 9 were expressed as albumin creatinine ratio (ACR) or albumin excretion rate (AER) and 3 reported as 24-h proteinuria or AER/Cr. In the random-effect model, the interventions had a significantly effect on albuminuria (Fig. 3C, 14 items, n = 321; SMD, − 1.60; 95% CI − 2.16 to − 1.04; *P* < 0.0001; *I*^2^ = 75%) compared to control.

##### Pathological changes in the kidney histology

Three studies [19, 23, 49] reported glomerular damage score found that the blockade or knockout of CB1 group could significantly reduce glomerular damage in renal dysfunction animals compared with the control group (Fig. 3D, 3 items, n = 87; SMD, − 1.28; 95% CI − 1.79 to − 0.77; *P* < 0.0001; *I*^2^ = 0%).

Three studies [21, 49, 51] reported tubular damage score found that the blockade or knockout of CB1 group could significantly reduce tubular damage in renal dysfunction animals compared with the control group (Fig. 3E, 4 items, n = 74; SMD, − 2.29; 95% CI − 3.56 to − 1.01; *P* = 0.0005; *I*^2^ = 68%).

##### Kidney weight/body weight ratio

A total of 8 included studies [20, 27, 30, 33, 41–43, 48] reported KW/ BW ratio after intervention with CB1 knockout or drug blockade. In the random-effect model, the blockade or knockout of CB1 had an uncertain effect on KW/ BW (Fig. 3F, 10 items, n = 186; SMD, − 0.06; 95% CI − 0.77 to 0.66; *P* = 0.88; *I*^2^ = 73%) compared to control.

#### Second outcomes

##### Blood glucose

**Fig. 4:**
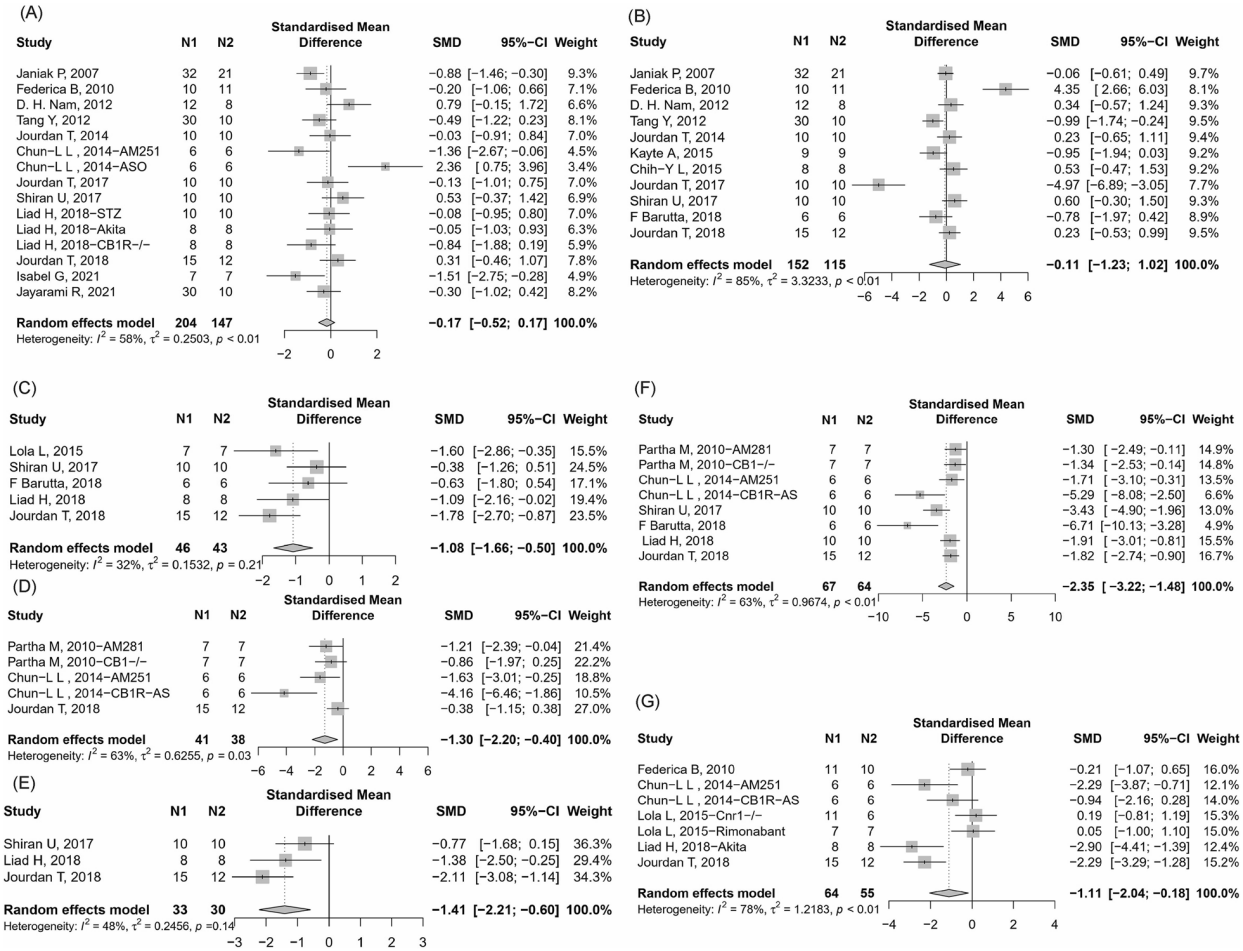
Meta-analysis and forest plot of effects of CB1 antagonist and knockout on second outcomes including blood glucose (**A**), body weight (**B**), metabolic and inflammatory related index (MCP-1, IL1β, IL18, TNF-α and TGF-β mRNA level, respectively) (**C**–**G**). N1 denotes the number of animals in the treatment group and N2 denotes the number of animals in the control group. The size of the square indicates the weight (%) of each study, the horizontal line shows the 95% confidence interval (95%-CI) of the individual standardized mean difference (SMD), and the black diamond represents the combined SMD and 95%-CI

A total of 12 included studies reported blood glucose level after intervention with CB1 genetic or drug blockade [19, 20, 27, 28, 30, 31, 39, 40, 42, 43, 49, 50]. In the random-effect model, the blockade or knockout of CB1 had an uncertain effect on blood glucose (Fig. 4A, 15 items, n = 351; SMD, − 0.17; 95% CI − 0.52 to 0.17; *P* = 0.33; *I*^2^ = 58%) compared to control.

##### Body weight

Eleven studies [19, 20, 27, 28, 30, 33, 35, 39–41, 43] reported tubular damage score found that the blockade or knockout of CB1 group could no significantly change body weight in animals compared with the control group (Fig. 4B, 11 items, n = 267; SMD, − 0.11; 95% CI − 1.23 to 1.02; *P* = 0.85; *I*^2^ = 85%).

##### Metabolic and inflammatory related index

Five studies [34, 40–43] reported MCP-1 mRNA level in renal found that the blockade or knockout of CB1 group could significantly reduce MCP-1 mRNA in renal tissue compared with the control group (Fig. 4C, 5 items, n = 89; SMD, − 1.08; 95% CI − 1.66 to − 0.50; *P* = 0.0003; *I*^2^ = 32%).

Three studies [21, 31, 43] reported IL1β mRNA level in renal found that the blockade or knockout of CB1 group could significantly reduce IL1β mRNA in renal tissue compared with the control group (Fig. 4D, 5 items, n = 79; SMD, − 1.30; 95% CI − 2.20 to − 0.40; *P* = 0.0047; *I*^2^ = 63%).

Three studies [40, 42, 43] reported IL18 mRNA level in renal found that the blockade or knockout of CB1 group could significantly reduce IL18 mRNA in renal tissue compared with the control group (Fig. 4E, 5 items, n = 63; SMD, − 1.41; 95% CI − 2.21 to − 0.60; *P* = 0.0006; *I*^2^ = 48%).

Six studies [21, 31, 40–43] reported TNF-α mRNA level in renal found that the blockade or knockout of CB1 group could significantly reduce TNF-α mRNA in renal tissue compared with the control group (Fig. 4F, 8 items, n = 131; SMD, − 2.35; 95% CI − 3.22 to − 1.48; *P* < 0.0001; *I*^2^ = 63%).

Five studies [20, 31, 34, 42, 43] reported TGF-β mRNA level in renal found that the blockade or knockout of CB1 group could significantly reduce TGF-β mRNA in renal tissue compared with the control group (Fig. 4G, 7 items, n = 119; SMD, − 1.11; 95% CI − 2.04 to − 0.18; *P* = 0.019; *I*^2^ = 78%).

### Effect of CB2 agonist on renal function

#### Primary outcomes

##### BUN

**Fig. 5:**
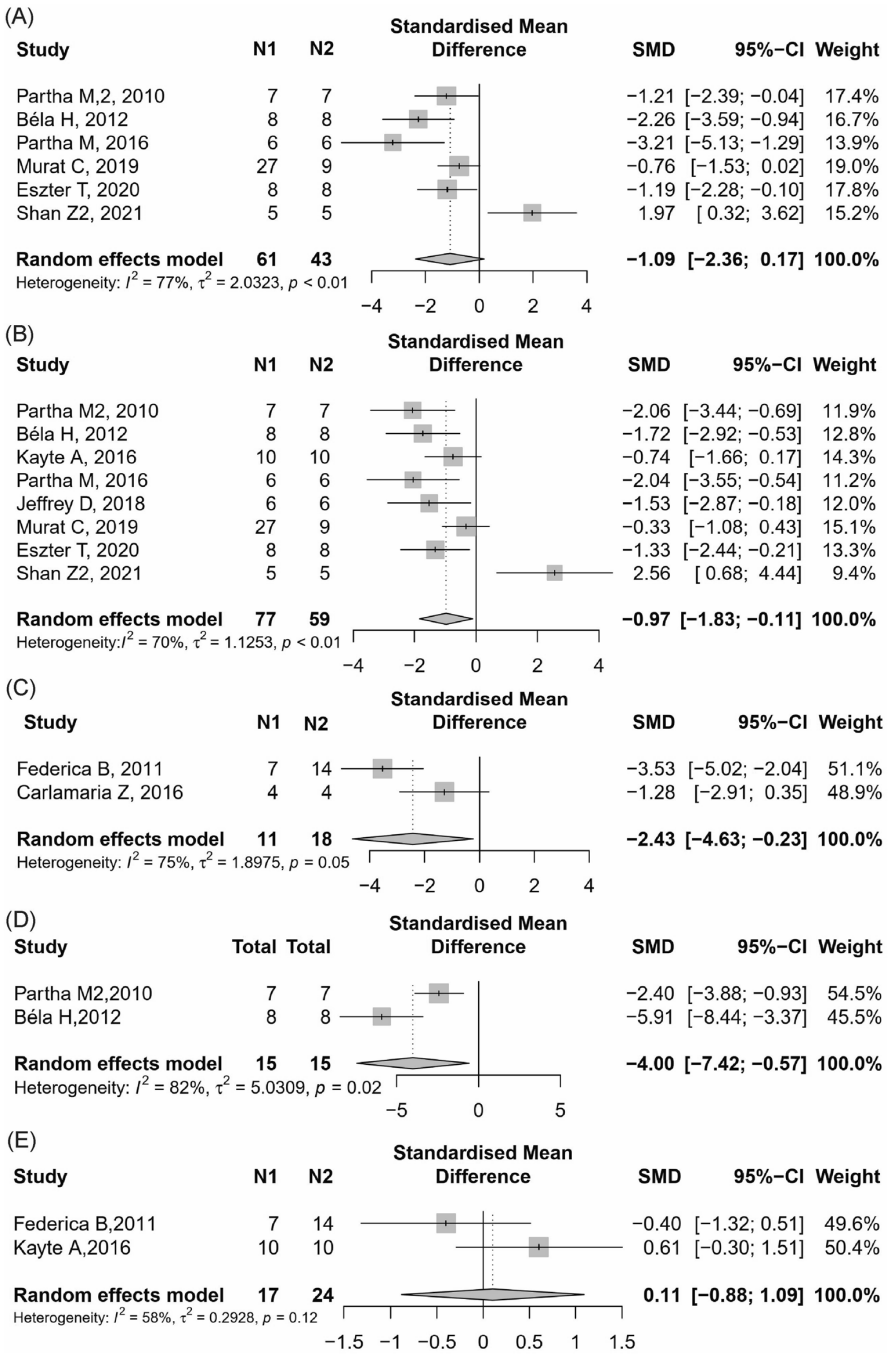
Meta-analysis and forest plot of effects of CB2 agonist on primary outcomes including blood urea nitrogen (**A**), serum creatinine (**B**), albuminuria (**C**), tubular damage score (**D**), and kidney weight/body weight ratio (**E**). N1 denotes the number of animals in the treatment group and N2 denotes the number of animals in the control group. The size of the square indicates the weight (%) of each study, the horizontal line shows the 95% confidence interval (95%-CI) of the individual standardized mean difference (SMD), and the black diamond represents the combined SMD and 95%-CI

Six studies [22, 26, 37, 46, 47, 53] reported BUN found that the activation of CB2 group could no significantly change BUN in renal dysfunction animals compared with the control group (Fig. 5A, 6 items, n = 104; SMD,− 1.09; 95% CI − 2.36 to 0.17; *P* = 0.09; *I*^2^ = 77%).

##### Scr

Eight studies [22, 26, 36, 37, 44, 46, 47, 53] reported Scr found that the activation of CB2 group could significantly reduce Scr in renal dysfunction animals compared with the control group (Fig. 5B, 8 items, n = 136; SMD, − 0.97; 95% CI − 1.83 to − 0.11; *P* = 0.03; *I*^2^ = 70%).

##### Albuminuria

Two studies [24, 38] reported 24 h urine protein found that the activation of CB2 group could significantly reduce albuminuria in renal dysfunction animals compared with the control group (Fig. 5C, 2 items, n = 29; SMD, − 2.43; 95% CI − 4.63 to − 0.23; *P* = 0.03; *I*^2^ = 75%).

##### Pathological changes in the kidney histology

Two studies [22, 26] reported tubular damage score found that the agonist of CB2 group could significantly reduce tubular damage in renal tissue compared with the control group (Fig. 5D, 2 items, n = 30; SMD, − 4.00; 95% CI − 7.42 to − 0.57; *P* = 0.02; *I*^2^ = 82%).

##### Kidney weight / body weight ratio

Two studies [24, 36] reported KW/BW ratio found that the activation of CB2 group could no significantly change KW/BW compared with the control group (Fig. 5E, 2 items, n = 41; SMD, 0.11; 95% CI − 0.88 to 1.09; *P* = 0.83; *I*^2^ = 58%).

#### Second outcomes

##### Blood glucose

**Fig. 6:**
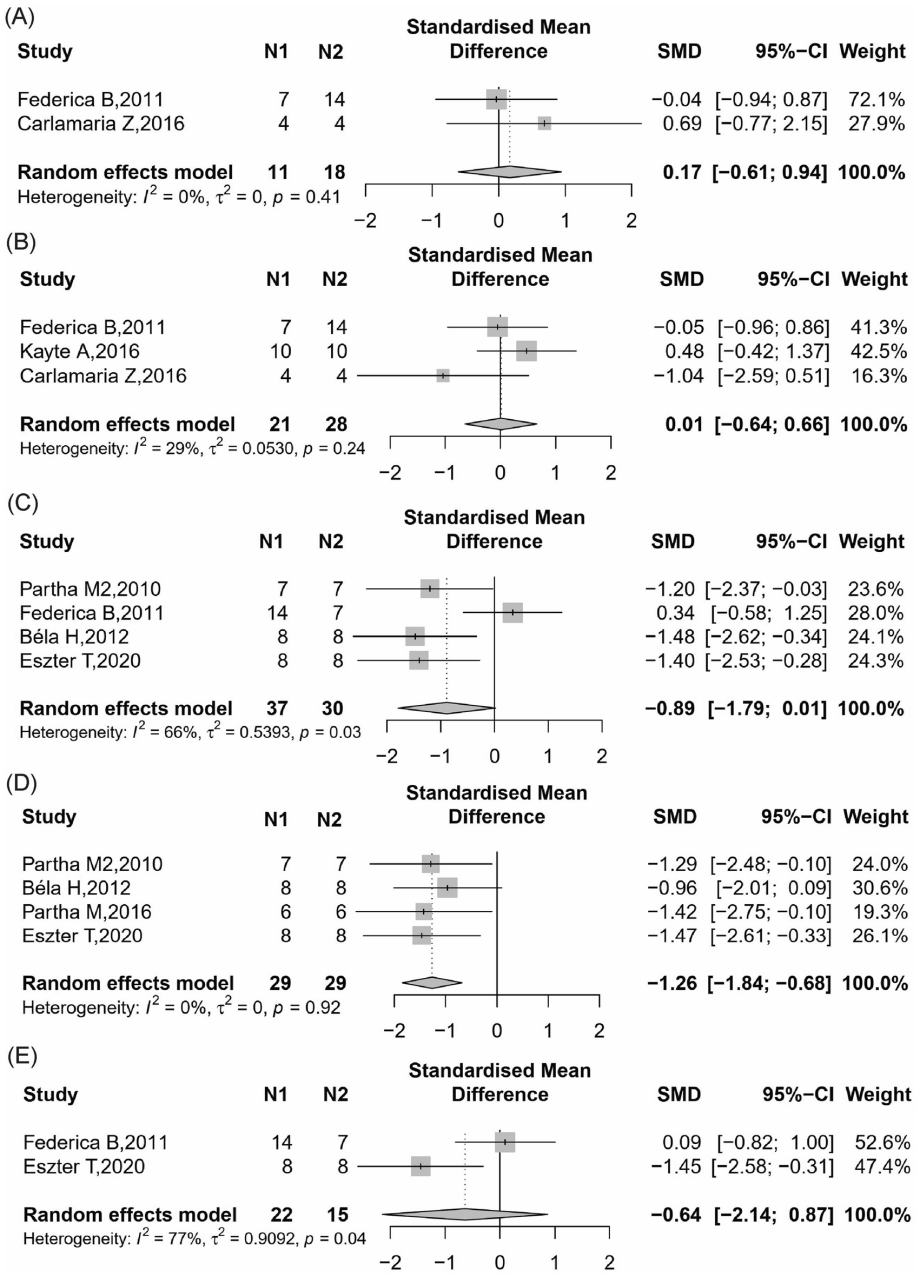
Meta-analysis and forest plot of effects of CB2 agonist on second outcomes including blood glucose (**A**), body weight (**B**), metabolic and inflammatory related index (MCP-1, TNF-α and TGF-β mRNA level, respectively) (**C**–**E**). N1 denotes the number of animals in the treatment group and N2 denotes the number of animals in the control group. The size of the square indicates the weight (%) of each study, the horizontal line shows the 95% confidence interval (95%-CI) of the individual standardized mean difference (SMD), and the black diamond represents the combined SMD and 95%-CI

A total of 2 included studies reported blood glucose level after intervention with CB2 agonist [24, 38]. In the random-effect model, the CB2 agonist had no effect on blood glucose (Fig. 6A, 2 items, n = 29; SMD, 0.17; 95% CI − 0.61 to 0.94; *P* = 0.67; *I*^2^ = 0%) compared to control.

##### Body weight

A total of 3 included studies reported body weight after intervention with CB2 agonist [24, 36, 38]. In the random-effect model, the CB2 agonist had no effect on body weight (Fig. 6B, 2 items, n = 49; SMD, 0.01; 95% CI − 0.64 to 0.66; *P* = 0.97; *I*^2^ = 29%) compared to control.

##### Metabolic and inflammatory related index

Four studies [22, 24, 26, 47] reported MCP-1 mRNA level in renal found that the activation of CB2 group could significantly reduce MCP-1 mRNA in renal tissue compared with the control group (Fig. 6C, 4 items, n = 58; SMD, − 1.26; 95% CI − 1.84 to − 0.68; *P* < 0.0001; *I*^2^ = 0%).

Four studies [22, 24, 37, 47] reported TNF-α mRNA level in renal found that the activation of CB2 group could significantly reduce TNF-α mRNA in renal tissue compared with the control group (Fig. 6D, 4 items, n = 58; SMD, − 1.26; 95% CI − 1.84 to − 0.68; *P* < 0.0001; *I*^2^ = 0%).

Two studies [24, 47] reported TGF-β mRNA level in renal found that the activation of CB2 group could no change TGF-β mRNA in renal tissue compared with the control group (Fig. 6E, 2 items, n = 37; SMD, − 0.64; 95% CI − 2.14 to 0.87; *P* = 0.41; *I*^2^ = 77%).

### Effect of CB2 antagonist and knockout on renal function

#### Primary outcomes

##### BUN

**Fig. 7:**
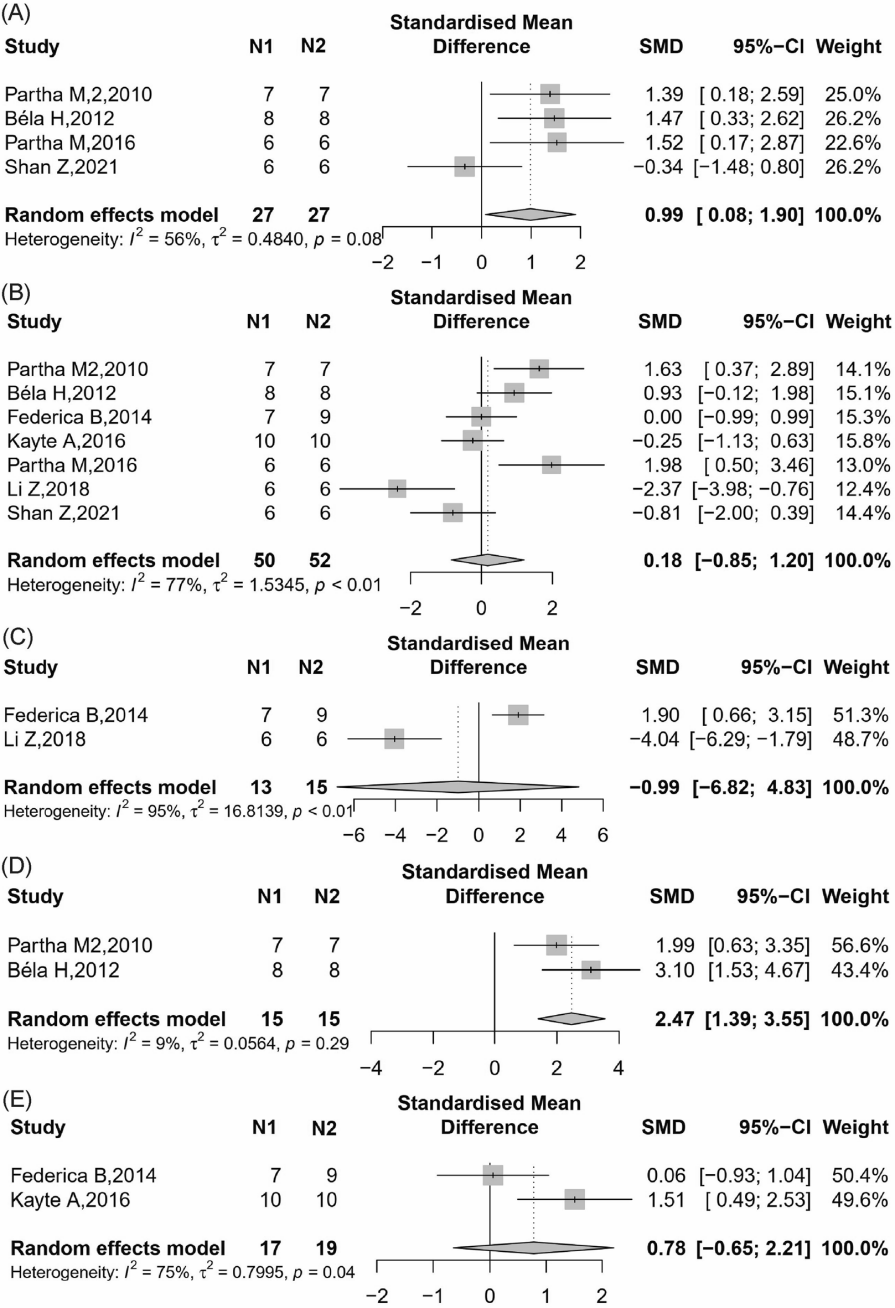
Meta-analysis and forest plot of effects of CB2 antagonist and knockout on primary outcomes including blood urea nitrogen (**A**), serum creatinine (**B**), albuminuria (**C**), tubular damage score (**D**) and kidney weight / body weight ratio (**E**). N1 denotes the number of animals in the treatment group and N2 denotes the number of animals in the control group. The size of the square indicates the weight (%) of each study, the horizontal line shows the 95% confidence interval (95%-CI) of the individual standardized mean difference (SMD), and the black diamond represents the combined SMD and 95%-CI

Four studies [22, 26, 37, 52] reported BUN found that the knockout or blockade of CB2 group could little or no change BUN in renal dysfunction animals compared with the control group (Fig. 7A, 4 items, n = 54; SMD, 0.99; 95% CI 0.08 to 1.90; *P* = 0.03; *I*^2^ = 56%).

##### Scr

Seven studies [22, 26, 29, 36, 45, 52] reported Scr found that the knockout or blockade of CB2 group had no effect on Scr in renal dysfunction animals compared with the control group (Fig. 7B, 7 items, n = 102; SMD, 0.18; 95% CI − 0.85 to 1.20; *P* = 0.73; *I*^2^ = 77%).

##### Albuminuria

Two studies [29, 45] reported ACR found that the blockade of CB2 group could no significantly change albuminuria in renal dysfunction animals compared with the control group (Fig. 7C, 2 items, n = 28; SMD, − 0.99; 95% CI − 6.82 to 4.83; *P* = 0.74; *I*^2^ = 95%).

##### Pathological changes in the kidney histology

Two studies [22, 26] reported tubular damage score found that the knockout of CB2 group could significantly increase tubular damage in renal tissue compared with the control group (Fig. 7D, 2 items, n = 30; SMD, 2.47; 95% CI 1.39 to 3.56; *P* < 0.0001; *I*^2^ = 9%).

##### Kidney weight / body weight ratio

Two studies [29, 36] reported KW/BW ratio found that the blockade or knockout of CB2 group could no significantly change KW/BW compared with the control group (Fig. 7E, 2 items, n = 36; SMD, 0.78; 95% CI − 0.65 to 2.21; *P* = 0.28; *I*^2^ = 75%).

#### Second outcomes

##### Body weight

**Fig. 8:**
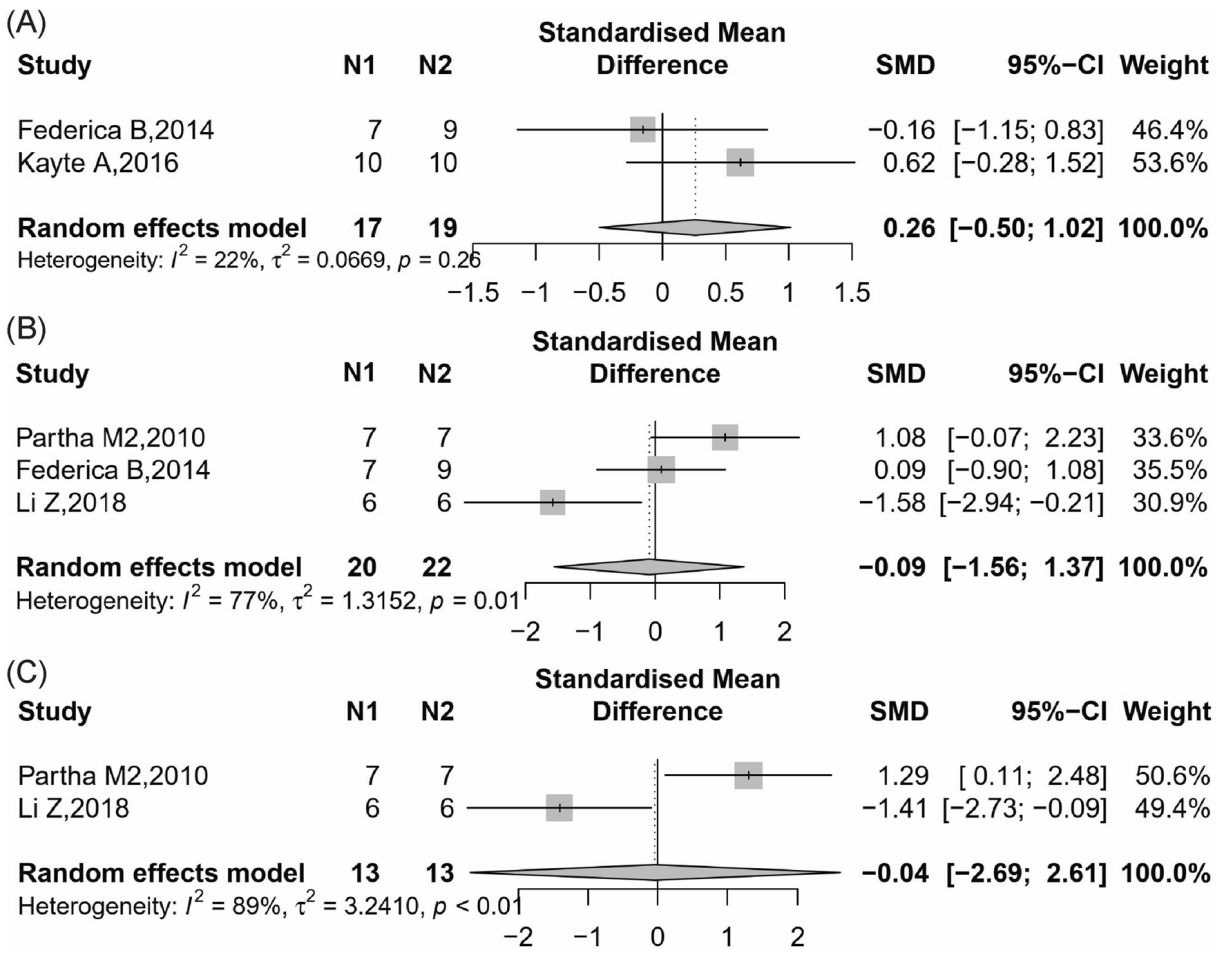
Meta-analysis and forest plot of effects of CB2 antagonist and knockout on second outcomes including body weight (**A**), and metabolic and inflammatory related index, MCP-1 (**B**) and TNF-α (**C**) mRNA level. N1 denotes the number of animals in the treatment group and N2 denotes the number of animals in the control group. The size of the square indicates the weight (%) of each study, the horizontal line shows the 95% confidence interval (95%-CI) of the individual standardized mean difference (SMD), and the black diamond represents the combined SMD and 95%-CI

A total of 2 included studies reported body weight after intervention with CB2 antagonist or knockout [29, 36]. In the random-effect model, the CB2 antagonist or knockout had no effect on body weight (Fig. 8A, 2 items, n = 36; SMD, 0.26; 95% CI − 0.50 to 1.02; *P* = 0.50; *I*^2^ = 22%) compared to control.

##### Metabolic and inflammatory related index

Three studies [22, 29, 45] reported MCP-1 mRNA level in renal found that the blockade or knockout of CB2 group could no change MCP-1 mRNA in renal tissue compared with the control group (Fig. 8B, 3 items, n = 42; SMD, − 0.09; 95% CI − 1.56 to 1.37; *P* = 0.90; *I*^2^ = 77%).

Two studies [22, 45] reported TNF-α mRNA level in renal found that the blockade or knockout of CB2 group could no change TNF-α mRNA in renal tissue compared with the control group (Fig. 8C, 2 items, n = 26; SMD, − 0.04; 95% CI − 2.69 to 2.61; *P* = 0.98; *I*^2^ = 89%).

### Effect of CB1 agonist on renal function

One study [32] reported that CB1 transgenes increased ACR (n = 20; SMD, 1.30; 95% CI 0.32 to 2.28; *P* = 0.0096) in mice, with no change blood glucose level (n = 20; SMD, − 0.44; 95% CI − 1.33 to 0.45; *P* = 0.33); as well as CB1 agonists increased ACR (n = 20; SMD, 1.06; 95% CI 0.11 to 2.01; *P* = 0.03) in rats, with no change blood glucose level (n = 20; SMD, 0.46; 95% CI − 0.43 to 1.35; *P* = 0.31).

Another study [31] reported that in mice CB1 transgenes increased IL1β mRNA expression (n = 12; SMD, 2.49; 95% CI 0.84 to 4.15; *P* = 0.0031) in kidney tissue.

### Publication bias

**Fig. 9:**
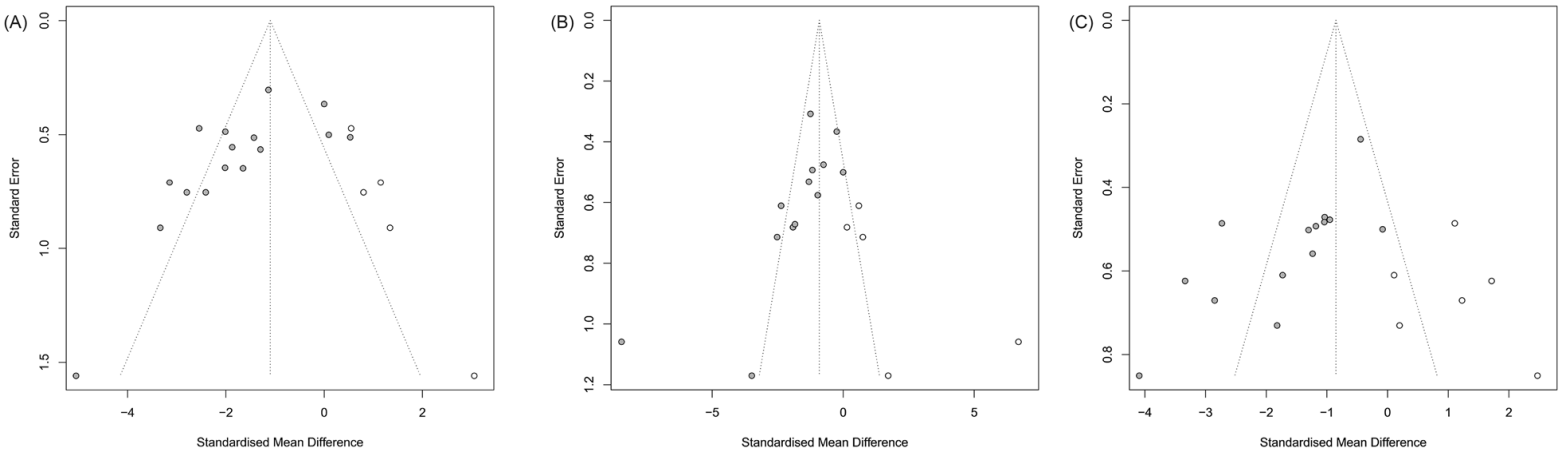
Funnel plots for CB1 antagonist and knockout on blood urea nitrogen (**A**), serum creatinine (**B**), and albuminuria (**C**) estimated by trim and fill analysis

Funnel plots and Egger’s test were constructed to evaluate the publication bias of the primary outcomes. Except for inhibitors of CB1 that could be included, other types were limited by the small number of studies (less than 10 studies). Bias was assessed by the trim and fill method. As funnel plots shown in Fig. 9 and Egger’ test in Table 3). Despite publication bias, BUN and ACR results were consistent after trimming and filling.

**Table 3:** Results from Egger's test and trim and fill analysis

Outcomes	No. of included items	SMD (95% CI)	*P* value	Egger's test	
				*t*	P value
(Before trim and fill)					
BUN	16	− 1.67[− 2.27,− 1.07]	< 0.001	− 2.82	0.014
Scr	13	− 1.88[− 2.91,− 0.85]	< 0.001	− 3.18	0.009
ACR	14	− 1.60[− 2.16,− 1.04]	< 0.001	− 3.66	0.003
(After trim and fill)					
BUN	21	− 1.10[− 1.79,− 0.41]	0.002	− 0.53	0.6
Scr	18	− 0.91[− 2.16, 0.33]	0.152	− 0.07	0.941
ACR	20	− 0.85[− 1.56,− 0.14]	0.019	− 0.32	0.756

### Sensitivity analysis* and subgroup analysis*

Sensitivity analysis was used to explore the impact of individual studies on the overall risk estimation, and the result shown in Additional file 1: Figs. S2, 3. Subgroup analyses were performed based on predefined classifications to explore possible sources of heterogeneity, and detailed results are shown in Additional file 1: Figs. S4–10.

#### CB1 antagonist and knockout primary outcomes

Sensitivity analysis shown that the pooled BUN, Scr and ACR results were stable when sequentially omitting one study in each iteration (Additional file 1: Fig. S2).

**Fig. 10:**
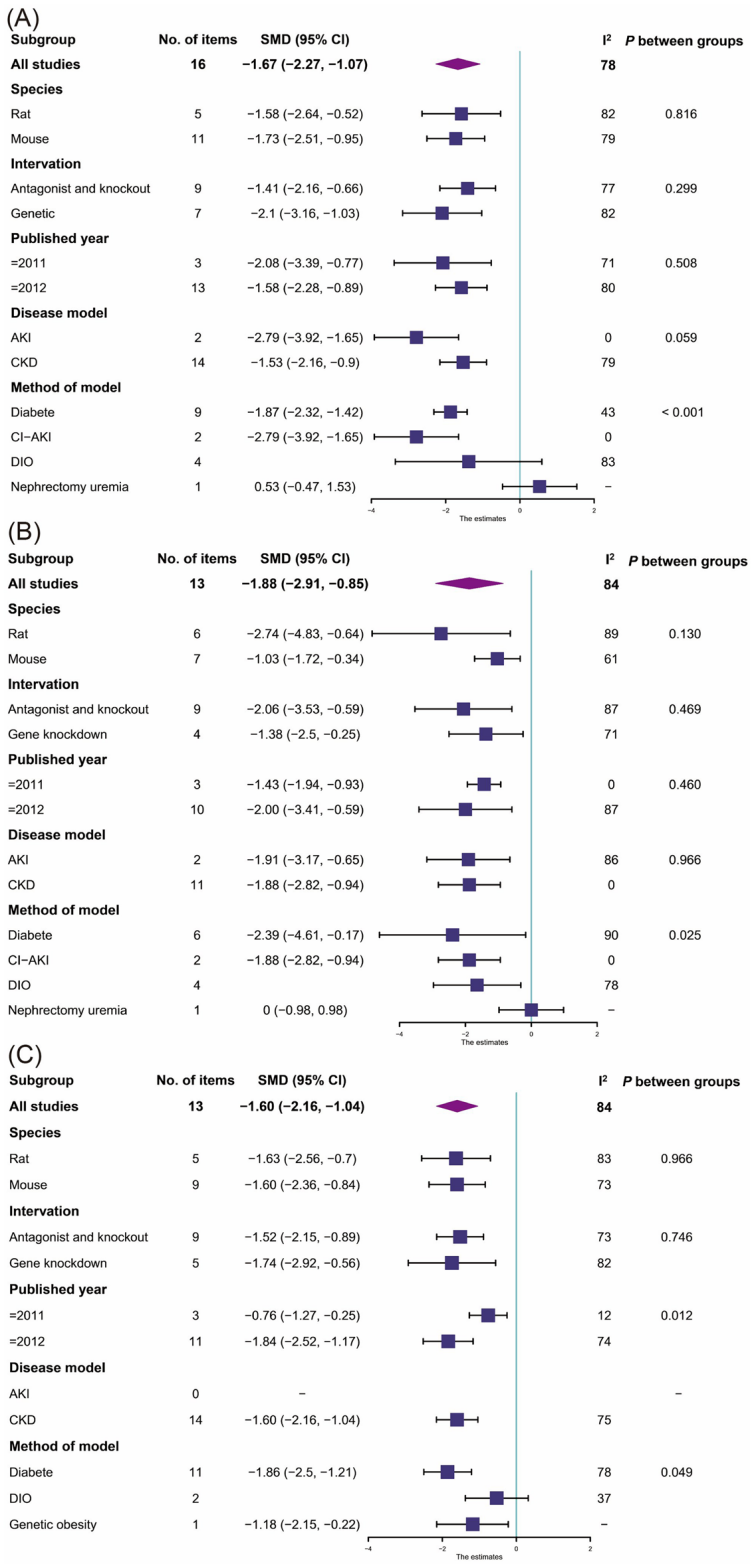
Forest plots for subgroup analyses of the CB1 antagonist and knockout primary outcomes including blood urea nitrogen (**A**), serum creatinine (**B**), and albuminuria (**C**)

In the analysis of subgroups, CB1 antagonist and knockout significantly reduced BUN, Scr and ACR, except in method of model subgroup (Fig. 10). Different approaches to disease modeling could be sources of heterogeneity.

#### CB2 agonist primary outcomes

Sensitivity shown that the combined BUN and Scr results were statistically significant after excluding the one study by Shan Z in 2021 (BUN, − 1.47 [− 2.19, − 0.76]; Scr, − 1.23 [− 1.78, − 0.68], Additional file 1: Fig. S3A, B).

**Fig. 11:**
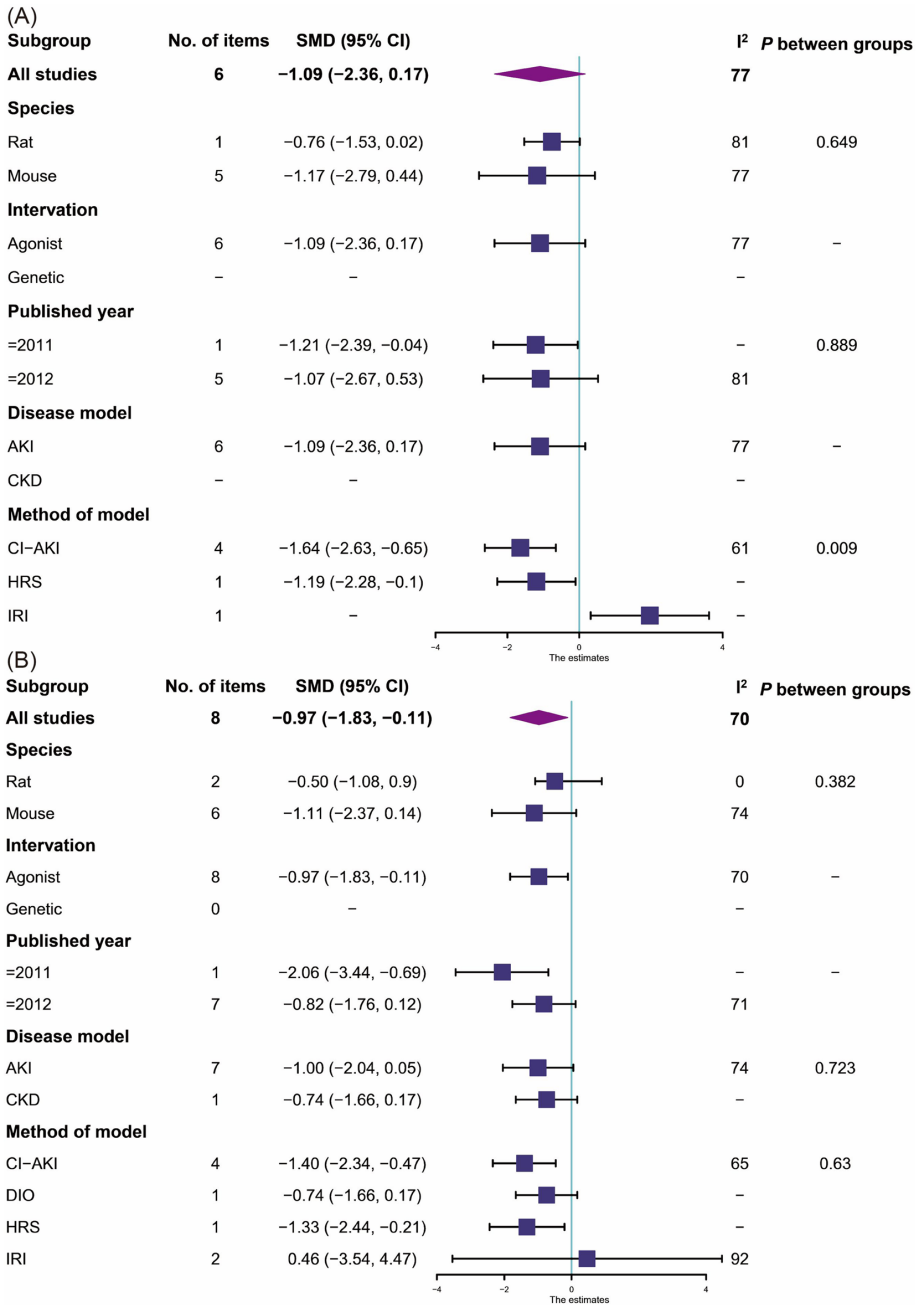
Forest plots for subgroup analyses of the CB2 agonist primary outcomes including blood urea nitrogen (**A**), and serum creatinine (**B**), and albuminuria (**C**)

In the analysis of subgroups, CB2 agonist could significantly reduce BUN and Scr in the CI-AKI subgroup (Fig. 11).

#### CB2 antagonist and knockout primary outcomes

Sensitivity shown that the pooled BUN and Scr results were stable when sequentially omitting one study in each iteration (Additional file 1: Figure S3C, D).

**Fig. 12:**
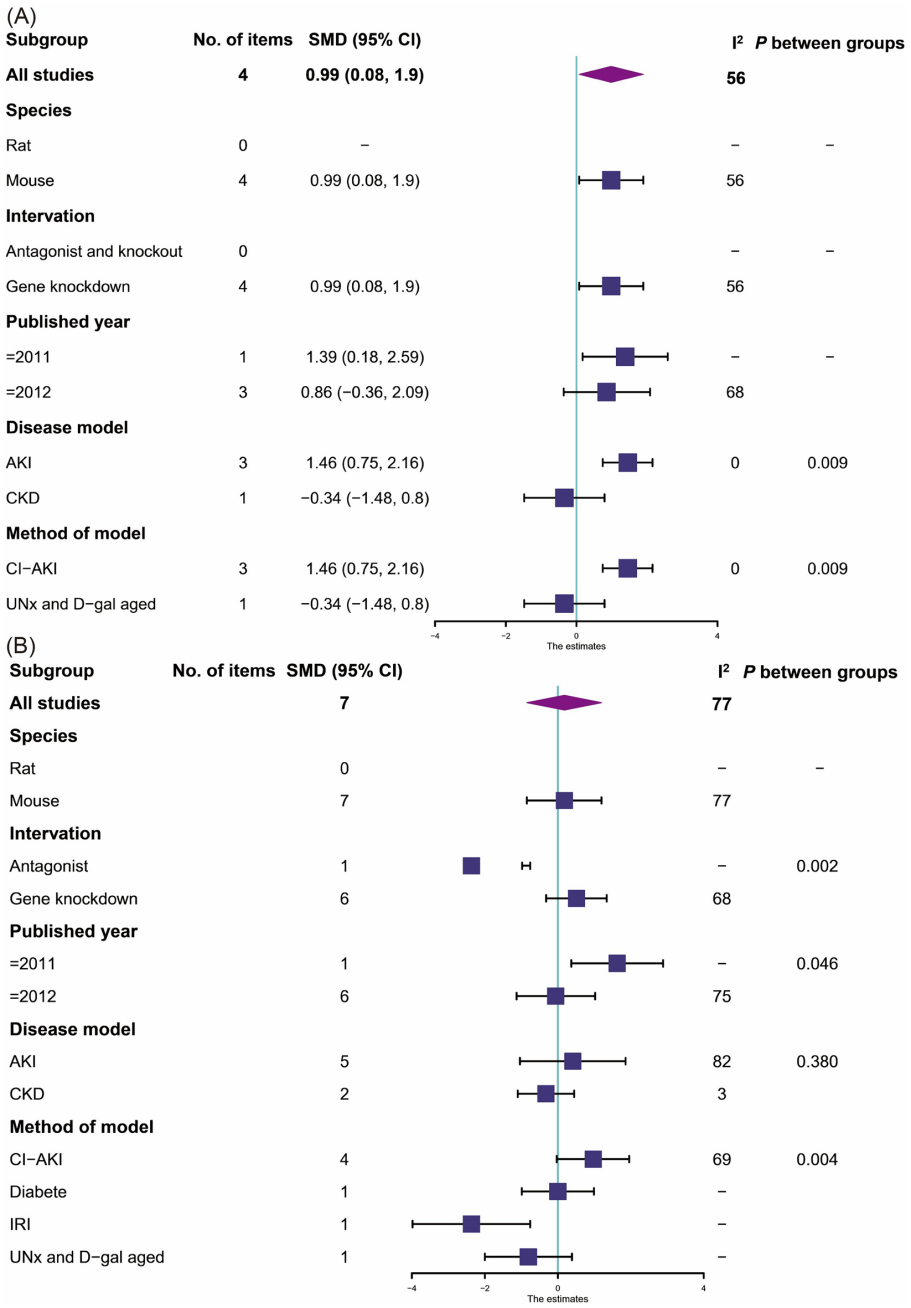
Forest plots for subgroup analyses of the CB2 antagonist and knockout Primary outcomes including blood urea nitrogen (**A**), and serum creatinine (**B**)

In the analysis of subgroups, CB2 antagonist could significantly reduce BUN and Scr in the CI-AKI subgroup (Fig. 12). Different approaches to disease modeling could be sources of heterogeneity.

## Discussion

### Summary of evidence and possible mechanisms

The present systematic review and meta-analysis provides a comprehensive synthesis of preclinical animal studies investigating the effects of cannabinoid receptor modulation on kidney disease. Our results indicate that both CB1 inhibition and CB2 receptor activation have reno-protective effects in animal models of kidney disease.

Firstly, the pooled analysis revealed potential reno-protective benefits of CB1 antagonists in animal models of kidney diseases, particularly in cases of diabetic nephropathy. In mouse models, CB1 antagonists or gene knockout significantly reduced urea nitrogen, creatinine, proteinuria, and pathological injury without affecting blood glucose levels, body weight or kidney/body weight ratio. Both sensitivity and subgroup analyses supported the consistent outcomes, although the heterogeneity could stem from the different disease modeling methods. Notably, CB1 antagonists and knockout effectively reduced BUN, Scr, and albuminuria, especially in diabetes cases. Furthermore, the decreased mRNA expression levels of inflammatory cytokines (including MCP-1, IL1β, IL18, TNF-α, and TGF-β) following CB1 antagonists treatment suggest potential anti-inflammatory effects. Inflammation plays a crucial role in the pathogenesis and progression of kidney disease by harming renal tissue, decreasing kidney function, and accelerating fibrosis. Conversely, kidney disease can lead to inflammation by activating immune cells and releasing pro-inflammatory cytokines [54–56]. Reducing inflammation can thus retard or halt the progression of kidney disease [55]. TGF-β, in particular, is the key mediator of renal inflammation and fibrosis [57, 58]. In response to injury or inflammation, TGF-β stimulates cells (including fibroblasts) in the kidney to produce extracellular matrix proteins, leading to scar tissue accumulation and fibrosis development [59, 60]. Additionally, TGF-β participates in immune cells' regulation and promotes pro-inflammatory cytokines and chemokines' production. Targeting TGF-β thus offers an attractive therapeutic intervention for renal inflammation and fibrosis [61–64]. By inhibiting TGF-β production, CB1 antagonists can prevent renal inflammation and fibrosis progression. These findings support previous studies demonstrating CB1 receptors' involvement in kidney disease's development and progression. Rimonabant, a representative CB1 antagonist [65–67], was approved by the European Medicines Agency in 2006 to reduce appetite via CB1 receptor antagonism in the brain, as an adjunct to diet and exercise in the treatment of obesity [68, 69]. Nonetheless, it was later withdrawn from the market due to higher risks of mental side effects (such as depression, anxiety, and suicidal thoughts), as clinical trials showed [70, 71].Recent years have seen the development of various new peripheral restricted CB1 inhibitors [72–74] (such as SLV319, JD5037, and AM6545, inclusive in this study), selectively inhibiting CB1 receptor activity in peripheral tissues to minimize brain exposure, while preserving mammalian receptor affinity and selectivity, potentially offering benefits in managing complications [75]. Notably, the researchers from the literature included in this study rarely added side effects such as mental status and cardiovascular events in evaluating cannabinoid receptors related to renal function in animals.

On the other hand, our analysis showed that CB2 agonists decreased creatinine and proteinuria in mice, while having no significant effect on urea nitrogen, blood glucose, body weight, and kidney to body weight ratio. Additionally, the use of CB2 agonists resulted in a reduction of renal pathological damage and mRNA expression levels of MCP-1 and TNF-α, but not TGF-β in this present meta-analysis. However, our sensitivity analysis revealed that the study by Shan Zhou et al. in [52] affected the statistical significance of the combined urea and creatinine results. They established an IRI mouse model by initial clipping of the left pedicle and unilateral nephrectomy one day before sacrifice and found that using the CB2 agonist AM1241 activated β-actin, increased urea nitrogen, and creatinine in UIRI mice. This contradicts other previous studies, including three models of cisplatin-induced acute kidney injury [22, 26, 37], a model of hepatorenal syndrome [47], and a rat model of IRI [46], which demonstrated the reduction of blood urea nitrogen and serum creatinine by CB2 agonists. Additionally, another study using SMM-295 [44], a novel CB2 agonist, significantly reduced serum creatinine in mice with acute kidney injury. In contrast, AM1241 has been shown to reduce proteinuria in STZ-induced diabetic mice [24]. Four of these studies reported a decrease in MCP-1 and TNF-α mRNA in renal tissue, collectively suggesting a protective effect of CB2 agonists. Differences in test time points, drugs used, and animal models of renal injury may be the main reasons for the inconsistent results. Subgroup analyses of the primary outcome with CB1 agonists showed that the modeling method may be a potential source of heterogeneity, with a statistically significant reduction in both SCR and BUN observed only in the CI-AKI subgroup.

Our analysis also showed that the combined results of CB2 receptor antagonists were not statistically significant, except for a slight alteration of urea nitrogen. CB2 receptor antagonist or gene knockout did not significantly affect creatinine, proteinuria, kidney weight/body weight ratio, and the expression levels of inflammation-related MCP-1 and TNF-α mRNA in the kidney of animals with renal injury, except for a possible slight increase in urea nitrogen from the results of four studies combined, and a significant increase in renal tubular pathological injury score from the results of two studies combined. However, caution must be exercised when interpreting the results due to the bias associated with a small number of studies and the variable diversity of interventions. Moreover, genetic approaches are harder to evaluate compared to small molecule drug interventions. In contrast, the effects of CB1 agonists were rarely reported in the included studies, with only one study reporting that the overexpression of CB1 gene increased proteinuria [32] and another study reporting that it increased the level of IL1β mRNA in the kidney [31]. Such limited results might have been due to our inclusion and exclusion criteria and concerns about cannabinoid abuse [76, 77]. Δ9-tetrahydrocannabinol (THC), a representative agonist of CB1, was the first to be chemically characterized as cannabinoids [78, 79]. Cannabidiol was found to be non-psychotic, while THC is responsible for the psychoactive effects of cannabis. CB1 is probably the most abundant and widespread G protein-coupled receptor in the mammalian brain, which makes therapeutic use of THC in pathological conditions very difficult [80].

Systematic reviews have investigated the effects of the cannabinoid system on pain [81, 82], but to our best knowledge, no other studies have been published with a meta-analysis in kidney disease. The endocannabinoid system comprises cannabinoid receptors (CB1 and CB2), endocannabinoids (eCB), and enzymes that regulate eCB biosynthesis and degradation. Dysregulation of this system has been linked to various pathological conditions, including pain disorders, neurodegenerative diseases, and metabolic disorders [83]. eCB is a lipid molecule synthesized on demand in response to various stimuli and acts as a retrograde messenger to regulate neurotransmitter release, predominantly anandamide and 2-arachidonoylglycerol. The CB1 receptor was initially identified as responsible for the mental effects of THC, or marijuana, to which anandamide binds with higher affinity [84, 85]. Subsequent research has shown that its high expression in peripheral and central nervous system cells can cause neuropathic and inflammatory pain, whereas CB2 is more expressed in immune cells, playing an anti-inflammatory role [86–88]. A large number of preclinical studies have recently revealed that peripheral CB1 can promote energy storage, affect lipid metabolism and insulin sensitivity, and significantly contribute to the pathogenesis of obesity, metabolic syndrome, and diabetes [89–91]. In contrast, the pathological state of CB2 is mainly present in inflammatory cells, where their anti-inflammatory effect includes inhibition of cytokine release [92–95]. In humans, most newly published structural biology studies have revealed different crystal structures of human CB1 and CB2, and small molecule drugs that affect CB1 and CB2 in different binding modes [96–99]. Cryo-electron microscopy has revealed the possible existence of this opposite activation spectrum of CB2 antagonism/CB1 agonism, representing a yin/yang functional relationship of CB2/CB1 [99, 100]. Particularly, CB1 and CB2 have been found to be expressed in a variety of cells in human normal kidney samples [11, 101], and increased expression of CB1 in renal biopsy specimens has been demonstrated in many renal diseases, including IgA nephropathy, acute interstitial nephritis, diabetic nephropathy, obesity-related glomerulopathy, and focal segmental glomerulosclerosis [34, 48]. In contrast, CB2 is expressed in decreased levels in diabetic nephropathy; however, it is thought to be greatly enhanced in lupus nephritis, membranous nephropathy, amyloid nephropathy, and immunoglobulin A nephropathy compared to a weak signal in healthy kidneys [24, 53]. Several reviews have been conducted on the pharmacological effects and research progress of CB1 and CB2 [80, 102, 103], especially in renal diseases [12, 104–106]. In general, the endocannabinoid system is complex, and the development of synthetic multi-target drugs from the endocannabinoidome is a promising direction. However, there are few studies exploring the interaction between CB1 and CB2 subtypes in renal diseases. Previous studies suggest that CB1 blockers primarily act on metabolism, as evidenced by parallel improvements in body weight, blood pressure, lipids, and insulin resistance [11]. In our meta-analysis, animal model groups treated with CB1 antagonist or knockout did not show statistically significant changes in KW/BW, blood glucose, or body weight compared to control groups. However, preclinical studies reported in the included literature suggest that CB1, affects renin-angiotensin system activity [30], influences the dynamic translocation of glucose transporter 2 in proximal tubular cells and glucose reabsorption [42], regulates the liver kinase B1/AMP-activated protein kinase signaling pathway [40], and affects cytoskeleton, extracellular matrix, apoptosis, and inflammatory cytokine secretion in vivo and in vitro [27, 34, 43]. The conflicting results on the renal protective effects of CB2 agonists are the most intriguing aspect of our study, and further research is needed to confirm and explore the underlying causes and mechanisms.

### Limitations

Despite the increasing attention given to the limitations of animal welfare, methodological concerns, and reproducibility of preclinical animal research, preclinical efficacy testing through specific disease animal models remains an indispensable element in drug development [107–111]. In particular, various disease models for kidney disease have been summarized [112–114], each endeavoring to mimic the human disease model, although none of them can fully replicate it. Therefore, we included models of acute kidney disease, such as cisplatin-induced drug-induced AKI, ischemia–reperfusion AKI with vascular occlusion, unilateral ureteral obstruction AKI, and the unusual bile duct ligation hepatorenal syndrome-induced AKI. The chronic kidney disease models included diabetes, obesity-induced CKD, partial nephrectomy CKD simulation, and the infrequent chronic intermittent hypoxia-induced chronic kidney injury. Multiple disease models in mice and rats may create confounding factors that increase potential bias. Additionally, the methodological quality of studies in our systematic review faced analogous issues. Most studies lacked descriptions of allocation concealment, random housing, and blinding, which significantly elevated the risk of bias in the study. The existence of publication bias further accentuates the need to promote the prospective registration of animal experiments and greater acceptance of negative results. Another issue that cannot be overlooked is the difference in drug dosages and the timing of evaluation indicator detection, which holds great importance for future research. Thus, there are multiple limitations in our meta-analysis that we should acknowledge. The heterogeneity of animal models, dosages, and treatment regimens used across studies may have influenced the results. Furthermore, the extrapolation of findings from animal models to humans should be done with caution due to the possible species-specific differences in the effects of cannabinoid receptor modulation. Although the complexity and diversity of the mechanisms involved in the endocannabinoid system entail the potential for interventional drugs with multiple targets, many mature marketed drugs contain multiple drug targets [80], such as the recently demonstrated renal protective effect of sodium-glucose co-transporter-2 inhibitors [115], reinforcing the importance of dosage and cross-species studies.

### Conclusions

**Fig. 13:**
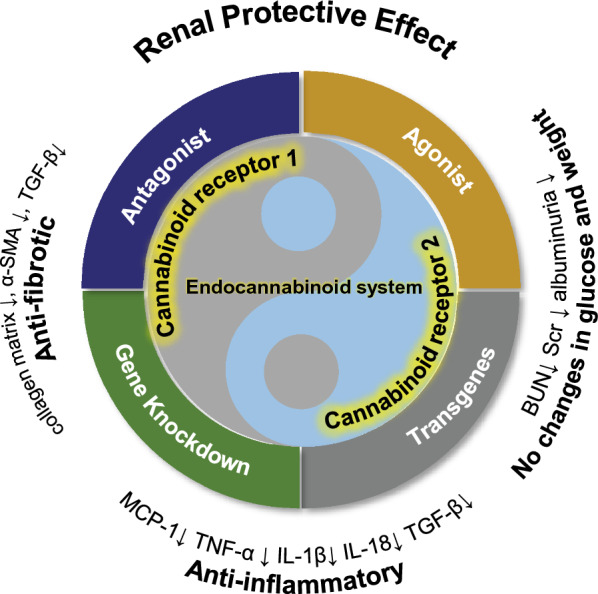
Schematic illustration of renal protection by intervention with CB1 and CB2. Pharmacological antagonism and genetic knockout of CB1, as well as pharmacological activation or genetic overexpression of CB2, may exert renal protective effects by downregulating TGF-β and α-SMA, and alleviating collagen matrix to exert anti-fibrotic effects. Additionally, by downregulating MCP-1, TNF-α, and interleukin factors, they act against inflammation. These mechanisms contribute to the reduction of markers like creatinine, urea nitrogen, and urinary protein in animal models of renal injury, without altering blood glucose levels or body weight. The regulatory effects of CB1 and CB2 on renal function can be likened to a "yin-yang" relationship

In conclusion, our study offers significant insights into the potential therapeutic effects of cannabinoid receptor modulation on kidney disease (Fig. 13). In particular, our results underscore the potential for CB1 inhibition and CB2 activation to mitigate kidney damage and inflammation. Future studies should seek to investigate the mechanisms of action of cannabinoid receptor modulation in kidney disease more comprehensively, along with the optimal dosages and treatment schedules for clinical use. It is worth further clinical inquiries into potential side effects and long-term safety of cannabinoid receptor modulation in animal models and eventually in human trials. Ultimately, our research contributes to the growing body of evidence that targeting cannabinoid receptors may be effective as a therapeutic approach to treating kidney disease.

### Supplementary Information

**Additional file 1: Figure S1.** Quality assessment graph of the included studies: reviewers’ judgments about each risk of bias item for eligible studies based on SYRCLE’s RoB tool for animal studies. **Figure S2.** Forest plot for sensitivity analysis on CB1 antagonist and knockout primary outcomes including blood urea nitrogen (A), serum creatinine (B) and albuminuria (C). **Figure S3.** Forest plot for sensitivity analysis on CB2 agonist primary outcomes including blood urea nitrogen (A) and serum creatinine (B); CB2 antagonist and knockout primary outcomes including blood urea nitrogen (C); and serum creatinine (D). **Figure S4.** Forest plots for subgroup analyses of the CB1 antagonist and knockout on blood urea nitrogen. Subgroup analyses were conducted stratified by the specie is rat or mouse (A); the intervention is antagonist or genetic (B); year of study published (C), (published = 1 means published in 2011 and earlier, published = 2 means published in 2012 and later); disease model is CKD or AKI (D); and method of model establishment is diabetes, cisplatin-induce AKI, DIO, or nephrectomy uremia (E). **Figure S5.** Forest plots for subgroup analyses of the CB1 antagonist and knockout on serum creatinine. Subgroup analyses were conducted stratified by the specie is rat or mouse (A); the intervention is antagonist or genetic (B); year of study published (C), (published = 1 means published in 2011 and earlier, published = 2 means published in 2012 and later); disease model is CKD or AKI (D); and method of model establishment is diabetes, cisplatin-induce AKI, DIO, or nephrectomy uremia (E). **Figure S6.** Forest plots for subgroup analyses of the CB1 antagonist and knockout on albuminuria. Subgroup analyses were conducted stratified by the specie is rat or mouse (A); the intervention is antagonist or genetic (B); year of study published (C), (published = 1 means published in 2011 and earlier, published = 2 means published in 2012 and later); disease model is CKD or AKI (D); and method of model establishment is diabetes or genetic obesity (E). **Figure S7.** Forest plots for subgroup analyses of the CB2 agonist on blood urea nitrogen. Subgroup analyses were conducted stratified by the specie is rat or mouse (A); the intervention is agonist or genetic (B); year of study published (C), (published = 1 means published in 2011 and earlier, published = 2 means published in 2012 and later); disease model is CKD or AKI (D); and method of model establishment is cisplatin-induce AKI, HRS or UUO (E). **Figure S8.** Forest plots for subgroup analyses of the CB2 agonist on serum creatinine. Subgroup analyses were conducted stratified by the specie is rat or mouse (A); the intervention is agonist or genetic (B); year of study published (C), (published = 1 means published in 2011 and earlier, published = 2 means published in 2012 and later); disease model is CKD or AKI (D); and method of model establishment is cisplatin-induce AKI, DIO, IRI or HRS (E). **Figure S9.** Forest plots for subgroup analyses of the CB2 antagonist and knockout on blood urea nitrogen. Subgroup analyses were conducted stratified by the specie is rat or mouse (A); the intervention is antagonist or gene knockout (B); year of study published (C), (published = 1 means published in 2011 and earlier, published = 2 means published in 2012 and later); disease model is CKD or AKI (D); and method of model establishment is cisplatin-induce AKI or unilateral nephrectomy and D-gal aged (E). **Figure S10.** Forest plots for subgroup analyses of the CB2 antagonist and knockout on serum creatinine. Subgroup analyses were conducted stratified by the specie is rat or mouse (A); the intervention is antagonist or gene knockout (B); year of study published (C), (published = 1 means published in 2011 and earlier, published = 2 means published in 2012 and later); disease model is CKD or AKI (D); and method of model establishment is cisplatin-induce AKI, DIO, IRI, or HRS (E).

## Data Availability

Data available on request from the authors.

## Abbreviations

ACR: Albumin Creatinine Ratio
AER: Albumin Excretion Rate
AKI: Acute Kidney Injury
BUN: Blood Urea Nitrogen
BW: Body Weight
CB1: Cannabinoid Receptor 1
CB2: Cannabinoid Receptor 2
CI: Confidence Interval
CI-AKI: Contrast-Induced Acute Kidney Injury
CKD: Chronic Kidney Disease
eCB: Endocannabinoids
IL 18: Interleukin 18
IL 1β: Interleukin-1 beta
IRI: Ischemia Reperfusion Injury
KW: Kidney Weight
MCP-1: Monocyte Chemotactic Protein 1
Scr: Serum Creatinine
SD: Standard Deviation
SEM: Standard Error of Mean
SMD: Standardized Mean Difference
STZ: Streptozotocin
TGF-β: Transforming Growth Factor-beta
THC: Δ9-Tetrahydrocannabinol
TNF-α: Tumor necrosis factor-alpha
UIRI: Unilateral Ischemia Reperfusion Injury
