# Supplementary material for: The intervention of cannabinoid receptor in chronic and acute kidney disease animal models: a systematic review and meta-analysis
Source: Diabetol Metab Syndr. 2024 Feb 15;16:45. doi: 10.1186/s13098-024-01283-2 (PMC10870675; doi:10.1186/s13098-024-01283-2)
Supplement: Supplementary file 1 — Additional file 1: Figure S1. Quality assessment graph of the included studies: reviewers’ judgments about each risk of bias item for eligible studies based on SYRCLE’s RoB tool for animal studies. Figure S2. Forest plot for sensitivity analysis on CB1 antagonist and knockout primary outcomes including blood urea nitrogen (A), serum creatinine (B) and albuminuria (C). Figure S3. Forest plot for sensitivity analysis on CB2 agonist primary outcomes including blood urea nitrogen (A) and serum creatinine (B); CB2 antagonist and knockout primary outcomes including blood urea nitrogen (C); and serum creatinine (D). Figure S4. Forest plots for subgroup analyses of the CB1 antagonist and knockout on blood urea nitrogen. Subgroup analyses were conducted stratified by the specie is rat or mouse (A); the intervention is antagonist or genetic (B); year of study published (C), (published = 1 means published in 2011 and earlier, published = 2 means published in 2012 and later); disease model is CKD or AKI (D); and method of model establishment is diabetes, cisplatin-induce AKI, DIO, or nephrectomy uremia (E). Figure S5. Forest plots for subgroup analyses of the CB1 antagonist and knockout on serum creatinine. Subgroup analyses were conducted stratified by the specie is rat or mouse (A); the intervention is antagonist or genetic (B); year of study published (C), (published = 1 means published in 2011 and earlier, published = 2 means published in 2012 and later); disease model is CKD or AKI (D); and method of model establishment is diabetes, cisplatin-induce AKI, DIO, or nephrectomy uremia (E). Figure S6. Forest plots for subgroup analyses of the CB1 antagonist and knockout on albuminuria. Subgroup analyses were conducted stratified by the specie is rat or mouse (A); the intervention is antagonist or genetic (B); year of study published (C), (published = 1 means published in 2011 and earlier, published = 2 means published in 2012 and later); disease model is CKD or AKI (D); and met [file 13098_2024_1283_MOESM1_ESM.zip › Supplemental Figure S1.pdf]

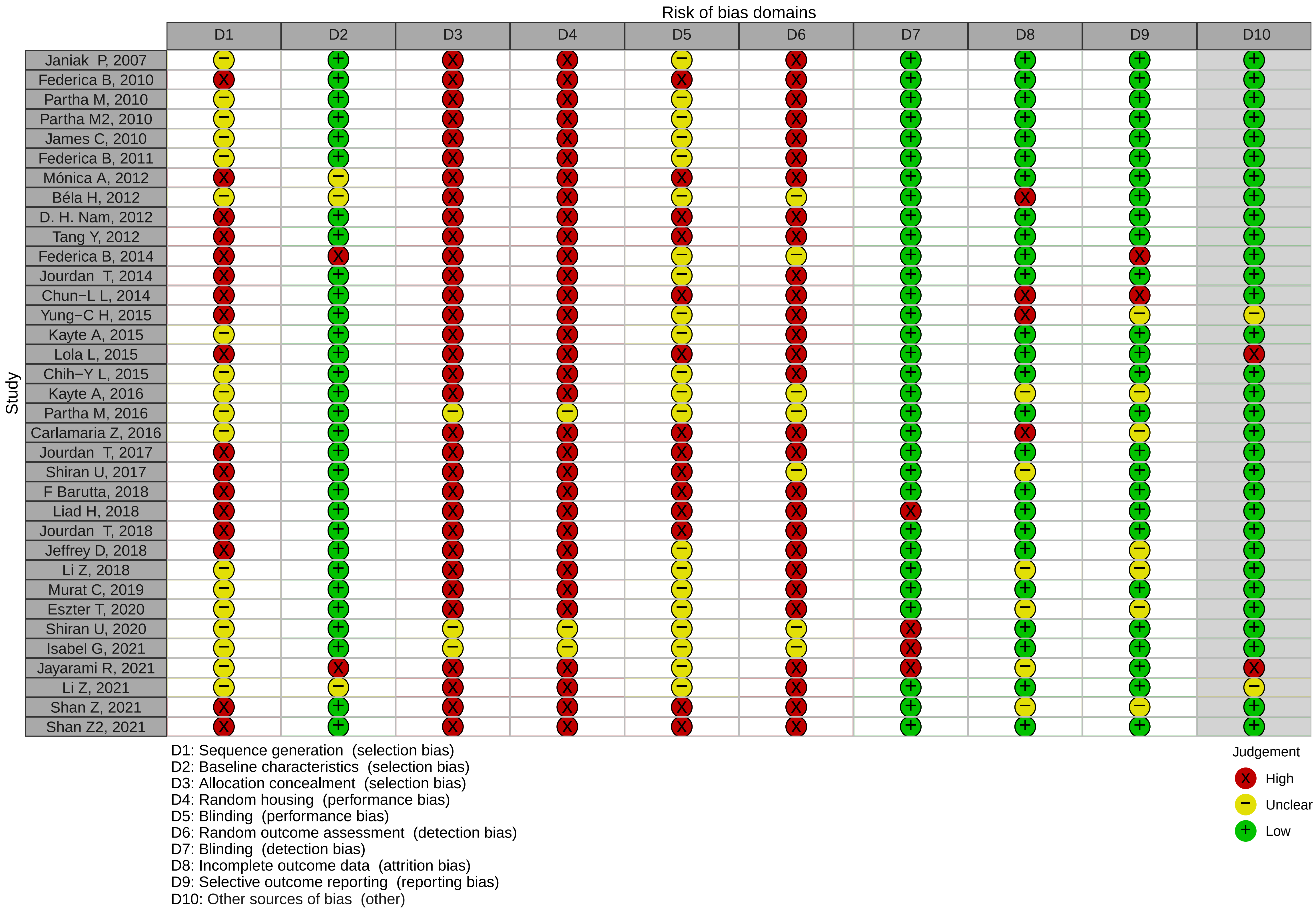
Risk of bias domains
D1
D2
D3
D4
D5
D6
D7
D8
D9
D10
Study
Janiak P, 2007
Federica B, 2010
Partha M, 2010
Partha M2, 2010
James C, 2010
Federica B, 2011
Mónica A, 2012
Béla H, 2012
D. H. Nam, 2012
Tang Y, 2012
Federica B, 2014
Jourdan T, 2014
Chun−L L, 2014
Yung−C H, 2015
Kayte A, 2015
Lola L, 2015
Chih−Y L, 2015
Kayte A, 2016
Partha M, 2016
Carlamaria Z, 2016
Jourdan T, 2017
Shiran U, 2017
F Barutta, 2018
Liad H, 2018
Jourdan T, 2018
Jeffrey D, 2018
Li Z, 2018
Murat C, 2019
Eszter T, 2020
Shiran U, 2020
Isabel G, 2021
Jayarami R, 2021
Li Z, 2021
Shan Z, 2021
Shan Z2, 2021
D1: Sequence generation (selection bias)
D2: Baseline characteristics (selection bias)
D3: Allocation concealment (selection bias)
D4: Random housing (performance bias)
D5: Blinding (performance bias)
D6: Random outcome assessment (detection bias)
D7: Blinding (detection bias)
D8: Incomplete outcome data (attrition bias)
D9: Selective outcome reporting (reporting bias)
D10: Other sources of bias (other)
Judgement
High
Unclear
Low
