# Supplementary material for: The intervention of cannabinoid receptor in chronic and acute kidney disease animal models: a systematic review and meta-analysis
Source: Diabetol Metab Syndr. 2024 Feb 15;16:45. doi: 10.1186/s13098-024-01283-2 (PMC10870675; doi:10.1186/s13098-024-01283-2)
Supplement: Supplementary file 1 — Additional file 1: Figure S1. Quality assessment graph of the included studies: reviewers’ judgments about each risk of bias item for eligible studies based on SYRCLE’s RoB tool for animal studies. Figure S2. Forest plot for sensitivity analysis on CB1 antagonist and knockout primary outcomes including blood urea nitrogen (A), serum creatinine (B) and albuminuria (C). Figure S3. Forest plot for sensitivity analysis on CB2 agonist primary outcomes including blood urea nitrogen (A) and serum creatinine (B); CB2 antagonist and knockout primary outcomes including blood urea nitrogen (C); and serum creatinine (D). Figure S4. Forest plots for subgroup analyses of the CB1 antagonist and knockout on blood urea nitrogen. Subgroup analyses were conducted stratified by the specie is rat or mouse (A); the intervention is antagonist or genetic (B); year of study published (C), (published = 1 means published in 2011 and earlier, published = 2 means published in 2012 and later); disease model is CKD or AKI (D); and method of model establishment is diabetes, cisplatin-induce AKI, DIO, or nephrectomy uremia (E). Figure S5. Forest plots for subgroup analyses of the CB1 antagonist and knockout on serum creatinine. Subgroup analyses were conducted stratified by the specie is rat or mouse (A); the intervention is antagonist or genetic (B); year of study published (C), (published = 1 means published in 2011 and earlier, published = 2 means published in 2012 and later); disease model is CKD or AKI (D); and method of model establishment is diabetes, cisplatin-induce AKI, DIO, or nephrectomy uremia (E). Figure S6. Forest plots for subgroup analyses of the CB1 antagonist and knockout on albuminuria. Subgroup analyses were conducted stratified by the specie is rat or mouse (A); the intervention is antagonist or genetic (B); year of study published (C), (published = 1 means published in 2011 and earlier, published = 2 means published in 2012 and later); disease model is CKD or AKI (D); and met [file 13098_2024_1283_MOESM1_ESM.zip › Supplementary-search strategy .docx]

**2021-08-20 Web of science**

#1 (TI=(Kidney OR renal OR Kidney disease OR Renal function OR kidney function tests OR gfr OR glomerular filtration rate OR scr OR cr OR ccr OR creatinine OR proteinuria OR urin$ protein excretion$ OR albuminuria OR AKI OR AIN OR CKD OR ESRD)) OR AB=(Kidney OR renal OR Kidney disease OR Renal function OR kidney function tests OR gfr OR glomerular filtration rate OR scr OR cr OR ccr OR creatinine OR proteinuria OR urin$ protein excretion$ OR albuminuria OR AKI OR AIN OR CKD OR ESRD) [758,370](https://www-webofscience-com-443.vpn.sicnu.edu.cn/wos/woscc/summary/f781ca77-5d60-4441-ba06-3ece7c75571c-04e9a851/relevance/1)

#2 (TI=(Cannabinoids OR cannabis OR marijuana OR marihuana OR hemp OR hashish OR cannabinoid OR cannabinoids OR cannabidiol OR tetrahydrocannabinol OR “endocannabinoid modulator” OR endocannabinoid OR endocannabinoids OR endo-cannabinoid OR nabilone OR Marinol OR levonantradol OR tetrahydrocannabinol OR cesamet OR nabiximols OR Sativex OR Cannabinoid Receptor Modulators OR Cannabinoid Receptor Agonists OR Cannabinoid Receptor Antagonists OR Receptors, Cannabinoid OR Receptor, Cannabinoid, CB1 OR Receptor, Cannabinoid, CB2 OR CB1R OR CB2R OR CBR1 OR CBR2 OR CBR OR CNR1 OR 2-AG OR AEA)) OR AB=(Cannabinoids OR cannabis OR marijuana OR marihuana OR hemp OR hashish OR cannabinoid OR cannabinoids OR cannabidiol OR tetrahydrocannabinol OR “endocannabinoid modulator” OR endocannabinoid OR endocannabinoids OR endo-cannabinoid OR nabilone OR Marinol OR levonantradol OR tetrahydrocannabinol OR cesamet OR nabiximols OR Sativex OR Cannabinoid Receptor Modulators OR Cannabinoid Receptor Agonists OR Cannabinoid Receptor Antagonists OR Receptors, Cannabinoid OR Receptor, Cannabinoid, CB1 OR Receptor, Cannabinoid, CB2 OR CB1R OR CB2R OR CBR1 OR CBR2 OR CBR OR CNR1 OR 2-AG OR AEA) [53,242](https://www-webofscience-com-443.vpn.sicnu.edu.cn/wos/woscc/summary/77453715-2af7-4a9d-a128-fa6122c3cbaa-04e9af74/relevance/1)

#3 (#1) AND #2 [769](https://www-webofscience-com-443.vpn.sicnu.edu.cn/wos/woscc/summary/84674d59-1401-4091-b69f-0f9dbf64d94e-04e9b3c3/relevance/1)

#4 (TI=(animals OR animal OR mice OR mus OR mouse OR murine OR woodmouse OR rats OR rat OR murinae OR muridae OR cottonrat OR cottonrats OR hamster OR hamsters OR cricetinae OR rodentia OR rodent OR rodents OR pigs OR pig OR swine OR swines OR piglets OR piglet OR boar OR boars OR "sus scrofa" OR ferrets OR ferret OR polecat OR polecats OR "mustela putorius" OR "guinea pigs" OR "guinea pig" OR cavia OR callithrix OR marmoset OR marmosets OR cebuella OR hapale OR octodon OR chinchilla OR chinchillas OR gerbillinae OR gerbil OR gerbils OR jird OR jirds OR merione OR meriones OR rabbits OR rabbit OR hares OR hare OR diptera OR flies OR fly OR dipteral OR drosphila OR drosophilidae OR cats OR cat OR carus OR felis OR nematoda OR nematode OR nematoda OR nematode OR nematodes OR sipunculida OR dogs OR dog OR canine OR canines OR canis OR sheep OR sheeps OR mouflon OR mouflons OR ovis OR goats OR goat OR capra OR capras OR rupicapra OR chamois OR haplorhini OR monkey OR monkeys OR anthropoidea OR anthropoids OR saguinus OR tamarin OR tamarins OR leontopithecus OR hominidae OR ape OR apes OR pan OR paniscus OR "pan paniscus" OR bonobo OR bonobos OR troglodytes OR "pan troglodytes" OR gibbon OR gibbons OR siamang OR siamangs OR nomascus OR symphalangus OR chimpanzee OR chimpanzees OR prosimians OR "bush baby" OR prosimian OR bush babies OR galagos OR galago OR pongidae OR gorilla OR gorillas OR pongo OR pygmaeus OR "pongo pygmaeus" OR orangutans OR pygmaeus OR lemur OR lemurs OR lemuridae OR horse OR horses OR pongo OR equus OR cow OR calf OR bull OR chicken OR chickens OR gallus OR quail OR bird OR birds OR quails OR poultry OR poultries OR fowl OR fowls OR reptile OR reptilia OR reptiles OR snakes OR snake OR lizard OR lizards OR alligator OR alligators OR crocodile OR crocodiles OR turtle OR turtles OR amphibian OR amphibians OR amphibia OR frog OR frogs OR bombina OR salientia OR toad OR toads OR "epidalea calamita" OR salamander OR salamanders OR eel OR eels OR fish OR fishes OR pisces OR catfish OR catfishes OR siluriformes OR arius OR heteropneustes OR sheatfish OR perch OR perches OR percidae OR perca OR trout OR trouts OR char OR chars OR salvelinus OR "fathead minnow" OR minnow OR cyprinidae OR carps OR carp OR zebrafish OR zebrafishes OR goldfish OR goldfishes OR guppy OR guppies OR chub OR chubs OR tinca OR barbels OR barbus OR pimephales OR promelas OR "poecilia reticulata" OR mullet OR mullets OR seahorse OR seahorse)) OR AB=(animals OR animal OR mice OR mus OR mouse OR murine OR woodmouse OR rats OR rat OR murinae OR muridae OR cottonrat OR cottonrats OR hamster OR hamsters OR cricetinae OR rodentia OR rodent OR rodents OR pigs OR pig OR swine OR swines OR piglets OR piglet OR boar OR boars OR "sus scrofa" OR ferrets OR ferret OR polecat OR polecats OR "mustela putorius" OR "guinea pigs" OR "guinea pig" OR cavia OR callithrix OR marmoset OR marmosets OR cebuella OR hapale OR octodon OR chinchilla OR chinchillas OR gerbillinae OR gerbil OR gerbils OR jird OR jirds OR merione OR meriones OR rabbits OR rabbit OR hares OR hare OR diptera OR flies OR fly OR dipteral OR drosphila OR drosophilidae OR cats OR cat OR carus OR felis OR nematoda OR nematode OR nematoda OR nematode OR nematodes OR sipunculida OR dogs OR dog OR canine OR canines OR canis OR sheep OR sheeps OR mouflon OR mouflons OR ovis OR goats OR goat OR capra OR capras OR rupicapra OR chamois OR haplorhini OR monkey OR monkeys OR anthropoidea OR anthropoids OR saguinus OR tamarin OR tamarins OR leontopithecus OR hominidae OR ape OR apes OR pan OR paniscus OR "pan paniscus" OR bonobo OR bonobos OR troglodytes OR "pan troglodytes" OR gibbon OR gibbons OR siamang OR siamangs OR nomascus OR symphalangus OR chimpanzee OR chimpanzees OR prosimians OR "bush baby" OR prosimian OR bush babies OR galagos OR galago OR pongidae OR gorilla OR gorillas OR pongo OR pygmaeus OR "pongo pygmaeus" OR orangutans OR pygmaeus OR lemur OR lemurs OR lemuridae OR horse OR horses OR pongo OR equus OR cow OR calf OR bull OR chicken OR chickens OR gallus OR quail OR bird OR birds OR quails OR poultry OR poultries OR fowl OR fowls OR reptile OR reptilia OR reptiles OR snakes OR snake OR lizard OR lizards OR alligator OR alligators OR crocodile OR crocodiles OR turtle OR turtles OR amphibian OR amphibians OR amphibia OR frog OR frogs OR bombina OR salientia OR toad OR toads OR "epidalea calamita" OR salamander OR salamanders OR eel OR eels OR fish OR fishes OR pisces OR catfish OR catfishes OR siluriformes OR arius OR heteropneustes OR sheatfish OR perch OR perches OR percidae OR perca OR trout OR trouts OR char OR chars OR salvelinus OR "fathead minnow" OR minnow OR cyprinidae OR carps OR carp OR zebrafish OR zebrafishes OR goldfish OR goldfishes OR guppy OR guppies OR chub OR chubs OR tinca OR barbels OR barbus OR pimephales OR promelas OR "poecilia reticulata" OR mullet OR mullets OR seahorse OR seahorse) [3,178,781](https://www-webofscience-com-443.vpn.sicnu.edu.cn/wos/woscc/summary/341641c7-8696-4bc4-ba32-9fa625648985-04e9bac2/relevance/1)

#5 (#3) AND #4 [235](https://www-webofscience-com-443.vpn.sicnu.edu.cn/wos/woscc/summary/bf8f9a3e-a06f-4f33-8c0f-d711ddf9c2c6-04e9bd5e/relevance/1)

**PUBMED**

(“Kidney disease” OR Renal function OR kidney function tests OR gfr OR glomerular filtration rate OR scr OR cr OR ccr OR creatinine OR proteinuria OR albuminuria OR urin$ protein excretion$ OR AKI OR AIN OR CKD OR ESRD) AND (Cannabinoids OR cannabis OR marijuana OR marihuana OR hemp OR hashish OR cannabinoid OR cannabinoids OR cannabidiol OR tetrahydrocannabinol OR “endocannabinoid modulator” OR endocannabinoid OR endocannabinoids OR endo-cannabinoid OR nabilone OR Marinol OR levonantradol OR tetrahydrocannabinol OR cesamet OR nabiximols OR Sativex OR Cannabinoid Receptor Modulators OR Cannabinoid Receptor Agonists OR Cannabinoid Receptor Antagonists OR Receptors, Cannabinoid OR Receptor, Cannabinoid, CB1 OR Receptor, Cannabinoid, CB2 OR CB1R OR CB2R OR CBR1 OR CBR2 OR CBR OR CNR1) 873

Animals 300

**EMBASE**

1. 'kidney disease'/exp OR 'renal function'/exp OR ' kidney function tests '/exp

2. $gfr OR 'glomerular filtration rate$' OR creatinine$ OR scr OR cr OR ccr

3. proteinuria$ OR 'urin$ protein excretion$' OR albuminuria$

4. 'acute kidney failure'/exp OR 'interstitial nephritis'/exp OR 'chronic kidney failure'/exp OR 'end stage renal disease'/exp

5. #1 OR #2 OR #3 OR #4

6. 'Cannabinoids'/exp OR 'cannabinoid receptor'/exp

7. cannabis OR marijuana OR marihuana OR hemp OR hashish OR cannabinoid OR cannabinoids OR cannabidiol OR tetrahydrocannabinol OR 'endocannabinoid modulator' OR endocannabinoid OR endocannabinoids OR 'endo-cannabinoid' OR nabilone OR Marinol OR levonantradol OR tetrahydrocannabinol OR cesamet OR nabiximols OR Sativex OR 'Cannabinoid Receptor Modulators' OR 'Cannabinoid Receptor Agonists' OR 'Cannabinoid Receptor Antagonists' OR 'Receptors, Cannabinoid' OR 'Receptor, Cannabinoid, CB1' OR 'Receptor, Cannabinoid, CB2'

8. #6 OR #7

9. #5 AND #8

#10 ND (**'animal experiment'**/de OR **'animal model'**/de OR **'animal tissue'**/de OR **'disease model'**/de OR **'in vivo study'**/de OR **'mouse model'**/de)
248

**#11**

**11 animal**:ab,ti OR **animals**:ab,ti OR **rodentia**:ab,ti OR **rodent**:ab,ti OR **rodents**:ab,ti OR **murinae**:ab,ti OR **mouse**:ab,ti OR **mice**:ab,ti OR **mus**:ab,ti OR **musculus**:ab,ti OR **murine**:ab,ti OR **woodmouse**:ab,ti OR **apodemus**:ab,ti OR **rat**:ab,ti OR **rats**:ab,ti OR **rattu**:ab,ti
314
