# Supplementary figures and images for: The intervention of cannabinoid receptor in chronic and acute kidney disease animal models: a systematic review and meta-analysis
Source: Diabetol Metab Syndr. 2024 Feb 15;16:45. doi: 10.1186/s13098-024-01283-2 (PMC10870675; doi:10.1186/s13098-024-01283-2)

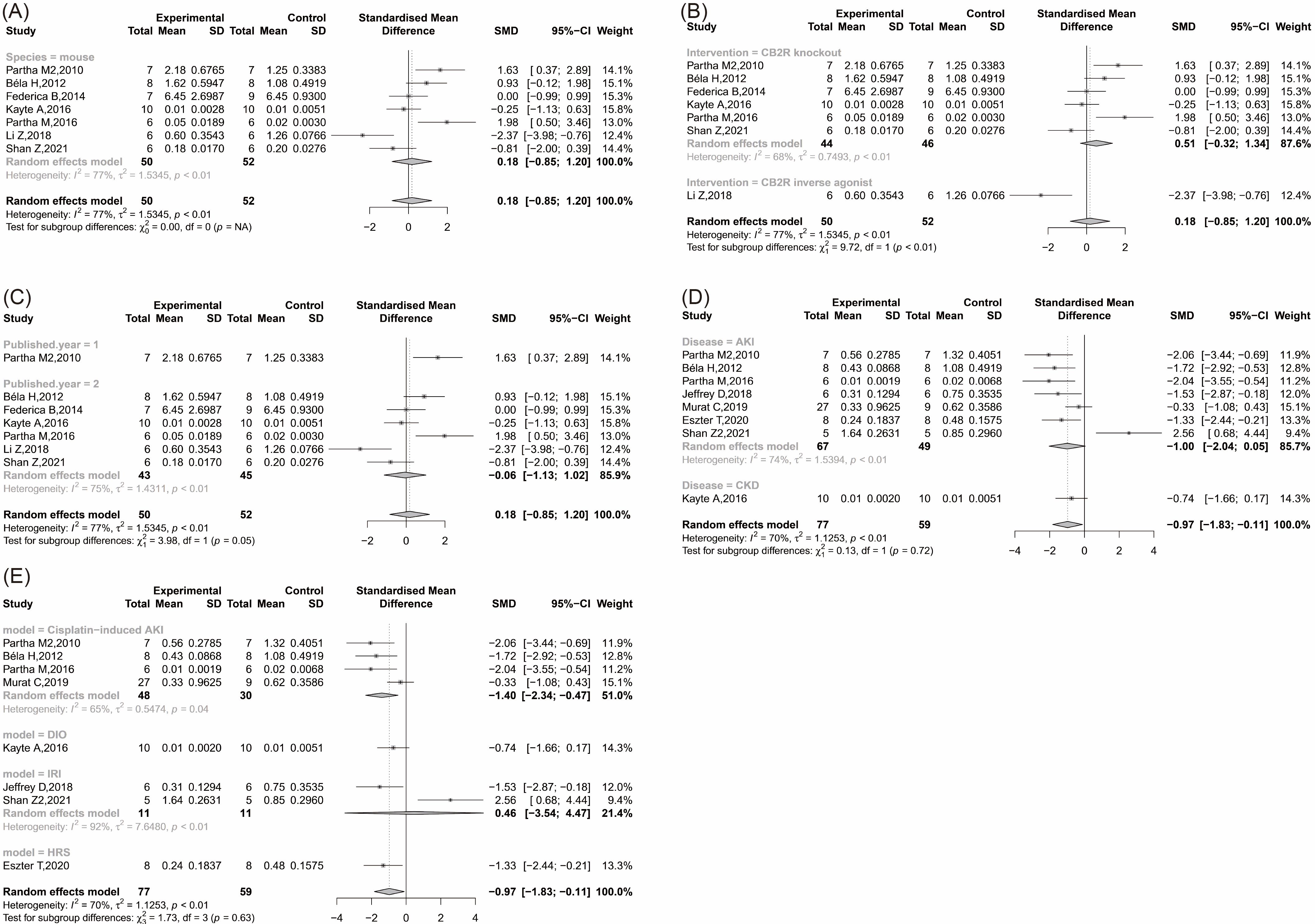

Supplement: Supplementary file 1 — Additional file 1: Figure S1. Quality assessment graph of the included studies: reviewers’ judgments about each risk of bias item for eligible studies based on SYRCLE’s RoB tool for animal studies. Figure S2. Forest plot for sensitivity analysis on CB1 antagonist and knockout primary outcomes including blood urea nitrogen (A), serum creatinine (B) and albuminuria (C). Figure S3. Forest plot for sensitivity analysis on CB2 agonist primary outcomes including blood urea nitrogen (A) and serum creatinine (B); CB2 antagonist and knockout primary outcomes including blood urea nitrogen (C); and serum creatinine (D). Figure S4. Forest plots for subgroup analyses of the CB1 antagonist and knockout on blood urea nitrogen. Subgroup analyses were conducted stratified by the specie is rat or mouse (A); the intervention is antagonist or genetic (B); year of study published (C), (published = 1 means published in 2011 and earlier, published = 2 means published in 2012 and later); disease model is CKD or AKI (D); and method of model establishment is diabetes, cisplatin-induce AKI, DIO, or nephrectomy uremia (E). Figure S5. Forest plots for subgroup analyses of the CB1 antagonist and knockout on serum creatinine. Subgroup analyses were conducted stratified by the specie is rat or mouse (A); the intervention is antagonist or genetic (B); year of study published (C), (published = 1 means published in 2011 and earlier, published = 2 means published in 2012 and later); disease model is CKD or AKI (D); and method of model establishment is diabetes, cisplatin-induce AKI, DIO, or nephrectomy uremia (E). Figure S6. Forest plots for subgroup analyses of the CB1 antagonist and knockout on albuminuria. Subgroup analyses were conducted stratified by the specie is rat or mouse (A); the intervention is antagonist or genetic (B); year of study published (C), (published = 1 means published in 2011 and earlier, published = 2 means published in 2012 and later); disease model is CKD or AKI (D); and met [file 13098_2024_1283_MOESM1_ESM.zip › Supplemental Figure S10.jpg]

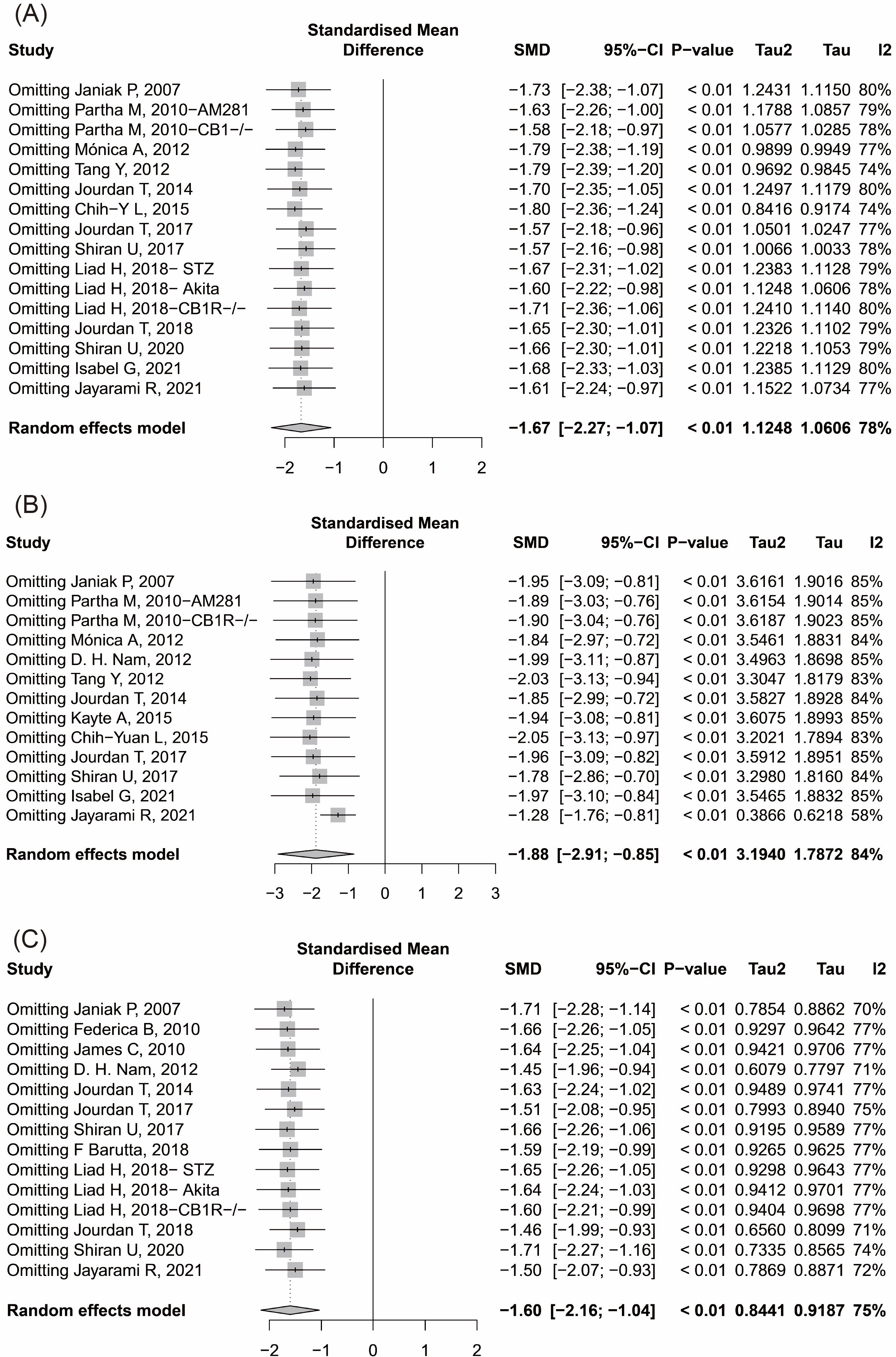

Supplement: Supplementary file 1 — Additional file 1: Figure S1. Quality assessment graph of the included studies: reviewers’ judgments about each risk of bias item for eligible studies based on SYRCLE’s RoB tool for animal studies. Figure S2. Forest plot for sensitivity analysis on CB1 antagonist and knockout primary outcomes including blood urea nitrogen (A), serum creatinine (B) and albuminuria (C). Figure S3. Forest plot for sensitivity analysis on CB2 agonist primary outcomes including blood urea nitrogen (A) and serum creatinine (B); CB2 antagonist and knockout primary outcomes including blood urea nitrogen (C); and serum creatinine (D). Figure S4. Forest plots for subgroup analyses of the CB1 antagonist and knockout on blood urea nitrogen. Subgroup analyses were conducted stratified by the specie is rat or mouse (A); the intervention is antagonist or genetic (B); year of study published (C), (published = 1 means published in 2011 and earlier, published = 2 means published in 2012 and later); disease model is CKD or AKI (D); and method of model establishment is diabetes, cisplatin-induce AKI, DIO, or nephrectomy uremia (E). Figure S5. Forest plots for subgroup analyses of the CB1 antagonist and knockout on serum creatinine. Subgroup analyses were conducted stratified by the specie is rat or mouse (A); the intervention is antagonist or genetic (B); year of study published (C), (published = 1 means published in 2011 and earlier, published = 2 means published in 2012 and later); disease model is CKD or AKI (D); and method of model establishment is diabetes, cisplatin-induce AKI, DIO, or nephrectomy uremia (E). Figure S6. Forest plots for subgroup analyses of the CB1 antagonist and knockout on albuminuria. Subgroup analyses were conducted stratified by the specie is rat or mouse (A); the intervention is antagonist or genetic (B); year of study published (C), (published = 1 means published in 2011 and earlier, published = 2 means published in 2012 and later); disease model is CKD or AKI (D); and met [file 13098_2024_1283_MOESM1_ESM.zip › Supplemental Figure S2.jpg]

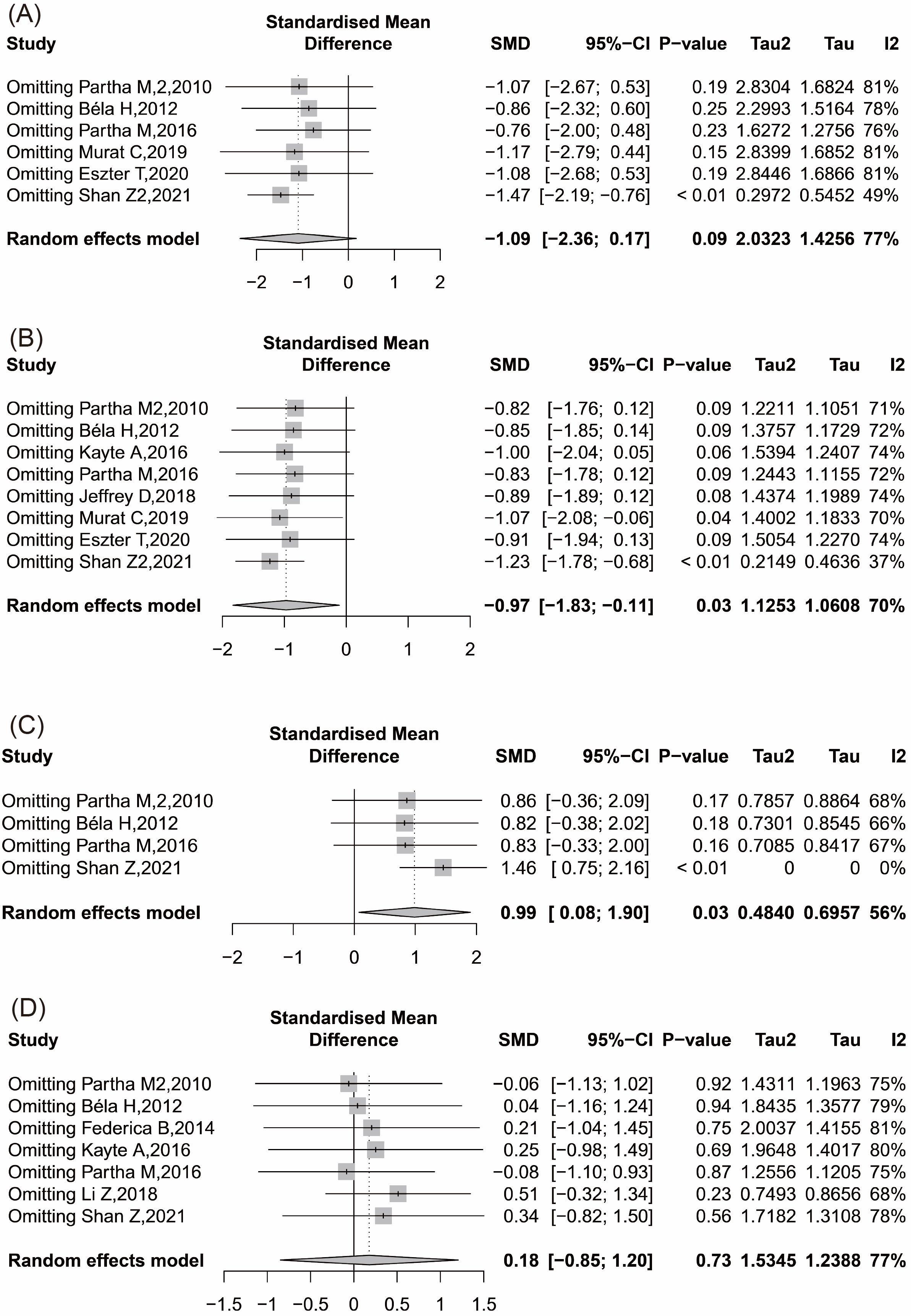

Supplement: Supplementary file 1 — Additional file 1: Figure S1. Quality assessment graph of the included studies: reviewers’ judgments about each risk of bias item for eligible studies based on SYRCLE’s RoB tool for animal studies. Figure S2. Forest plot for sensitivity analysis on CB1 antagonist and knockout primary outcomes including blood urea nitrogen (A), serum creatinine (B) and albuminuria (C). Figure S3. Forest plot for sensitivity analysis on CB2 agonist primary outcomes including blood urea nitrogen (A) and serum creatinine (B); CB2 antagonist and knockout primary outcomes including blood urea nitrogen (C); and serum creatinine (D). Figure S4. Forest plots for subgroup analyses of the CB1 antagonist and knockout on blood urea nitrogen. Subgroup analyses were conducted stratified by the specie is rat or mouse (A); the intervention is antagonist or genetic (B); year of study published (C), (published = 1 means published in 2011 and earlier, published = 2 means published in 2012 and later); disease model is CKD or AKI (D); and method of model establishment is diabetes, cisplatin-induce AKI, DIO, or nephrectomy uremia (E). Figure S5. Forest plots for subgroup analyses of the CB1 antagonist and knockout on serum creatinine. Subgroup analyses were conducted stratified by the specie is rat or mouse (A); the intervention is antagonist or genetic (B); year of study published (C), (published = 1 means published in 2011 and earlier, published = 2 means published in 2012 and later); disease model is CKD or AKI (D); and method of model establishment is diabetes, cisplatin-induce AKI, DIO, or nephrectomy uremia (E). Figure S6. Forest plots for subgroup analyses of the CB1 antagonist and knockout on albuminuria. Subgroup analyses were conducted stratified by the specie is rat or mouse (A); the intervention is antagonist or genetic (B); year of study published (C), (published = 1 means published in 2011 and earlier, published = 2 means published in 2012 and later); disease model is CKD or AKI (D); and met [file 13098_2024_1283_MOESM1_ESM.zip › Supplemental Figure S3.jpg]

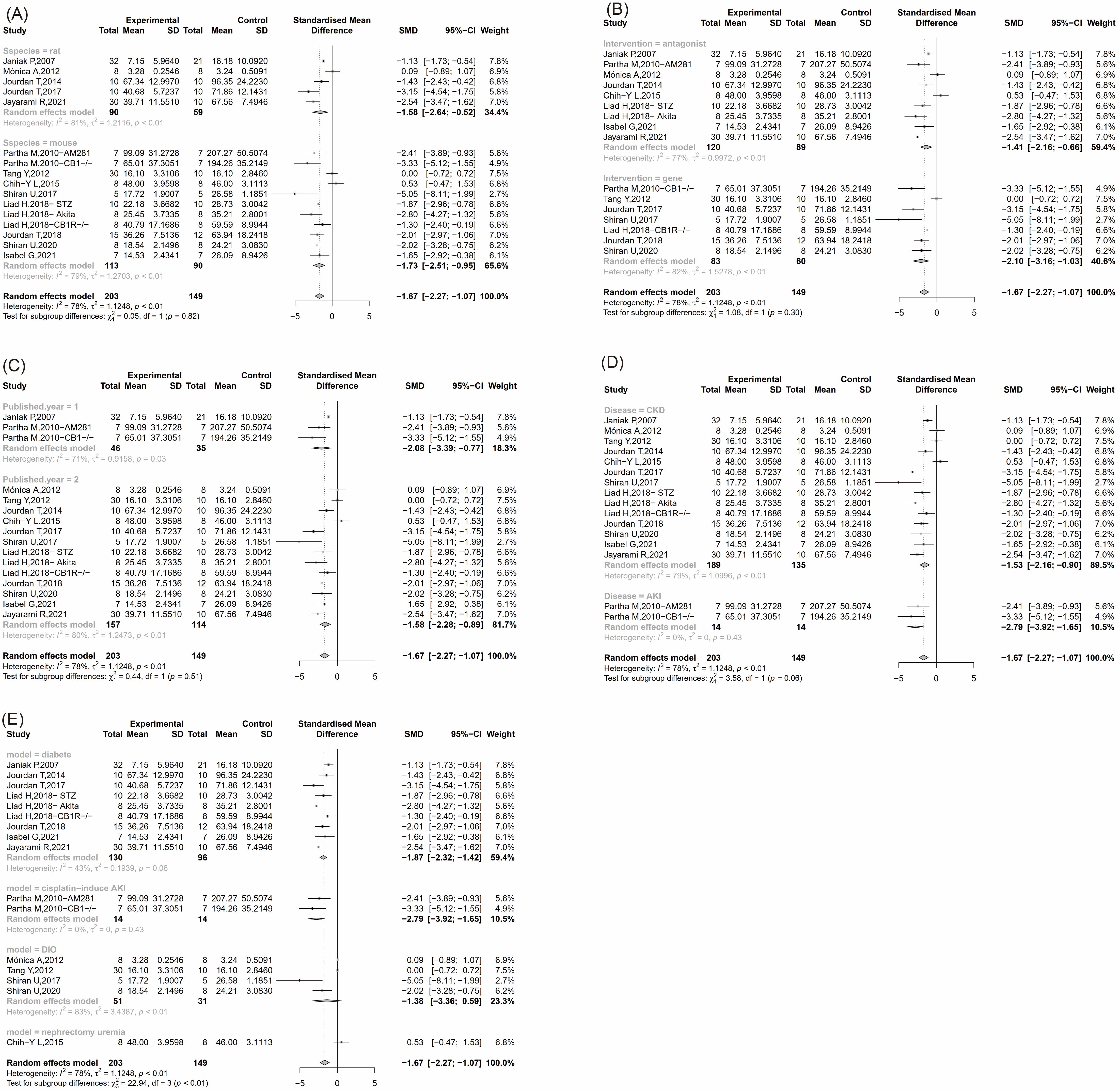

Supplement: Supplementary file 1 — Additional file 1: Figure S1. Quality assessment graph of the included studies: reviewers’ judgments about each risk of bias item for eligible studies based on SYRCLE’s RoB tool for animal studies. Figure S2. Forest plot for sensitivity analysis on CB1 antagonist and knockout primary outcomes including blood urea nitrogen (A), serum creatinine (B) and albuminuria (C). Figure S3. Forest plot for sensitivity analysis on CB2 agonist primary outcomes including blood urea nitrogen (A) and serum creatinine (B); CB2 antagonist and knockout primary outcomes including blood urea nitrogen (C); and serum creatinine (D). Figure S4. Forest plots for subgroup analyses of the CB1 antagonist and knockout on blood urea nitrogen. Subgroup analyses were conducted stratified by the specie is rat or mouse (A); the intervention is antagonist or genetic (B); year of study published (C), (published = 1 means published in 2011 and earlier, published = 2 means published in 2012 and later); disease model is CKD or AKI (D); and method of model establishment is diabetes, cisplatin-induce AKI, DIO, or nephrectomy uremia (E). Figure S5. Forest plots for subgroup analyses of the CB1 antagonist and knockout on serum creatinine. Subgroup analyses were conducted stratified by the specie is rat or mouse (A); the intervention is antagonist or genetic (B); year of study published (C), (published = 1 means published in 2011 and earlier, published = 2 means published in 2012 and later); disease model is CKD or AKI (D); and method of model establishment is diabetes, cisplatin-induce AKI, DIO, or nephrectomy uremia (E). Figure S6. Forest plots for subgroup analyses of the CB1 antagonist and knockout on albuminuria. Subgroup analyses were conducted stratified by the specie is rat or mouse (A); the intervention is antagonist or genetic (B); year of study published (C), (published = 1 means published in 2011 and earlier, published = 2 means published in 2012 and later); disease model is CKD or AKI (D); and met [file 13098_2024_1283_MOESM1_ESM.zip › Supplemental Figure S4.jpg]

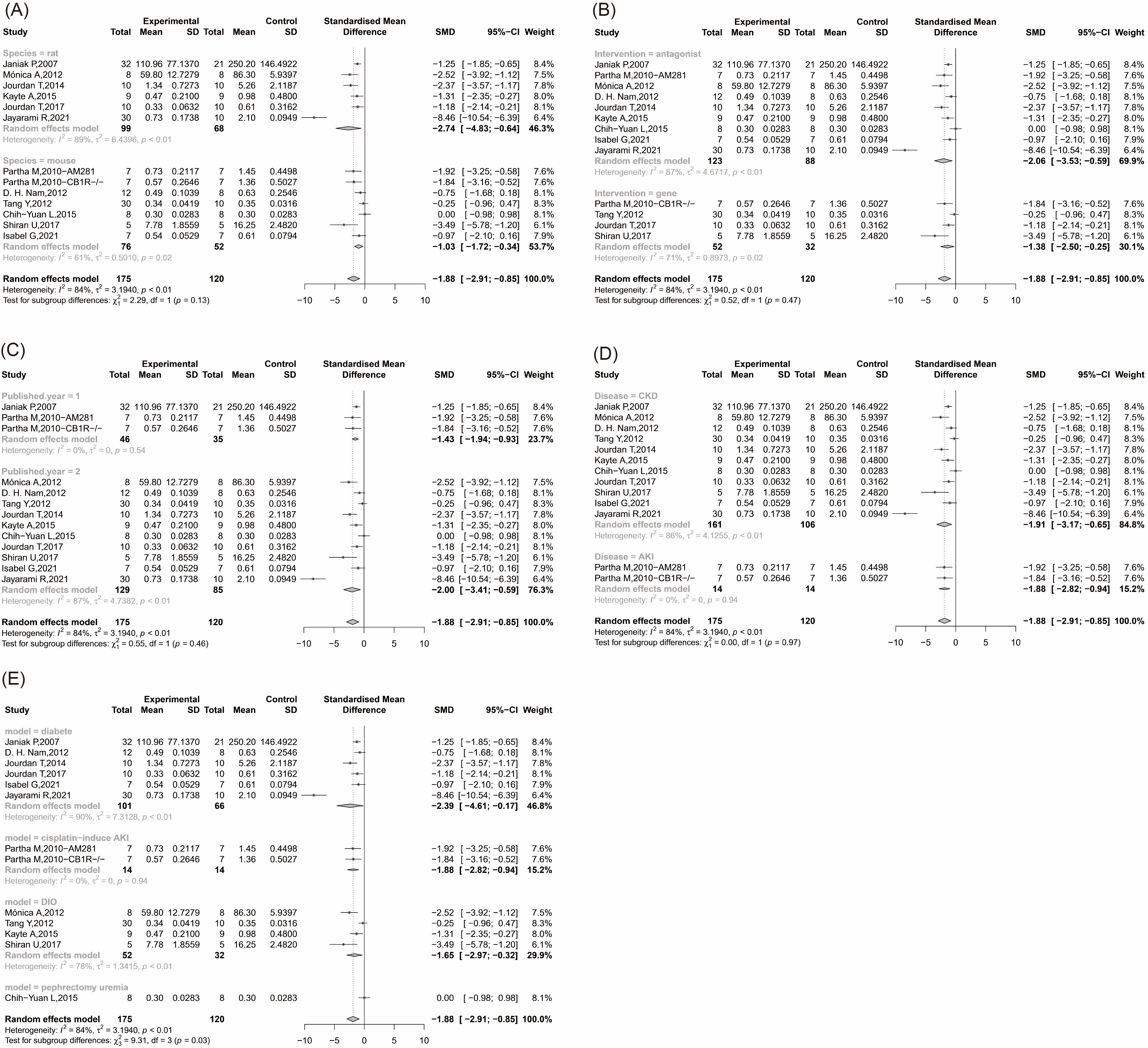

Supplement: Supplementary file 1 — Additional file 1: Figure S1. Quality assessment graph of the included studies: reviewers’ judgments about each risk of bias item for eligible studies based on SYRCLE’s RoB tool for animal studies. Figure S2. Forest plot for sensitivity analysis on CB1 antagonist and knockout primary outcomes including blood urea nitrogen (A), serum creatinine (B) and albuminuria (C). Figure S3. Forest plot for sensitivity analysis on CB2 agonist primary outcomes including blood urea nitrogen (A) and serum creatinine (B); CB2 antagonist and knockout primary outcomes including blood urea nitrogen (C); and serum creatinine (D). Figure S4. Forest plots for subgroup analyses of the CB1 antagonist and knockout on blood urea nitrogen. Subgroup analyses were conducted stratified by the specie is rat or mouse (A); the intervention is antagonist or genetic (B); year of study published (C), (published = 1 means published in 2011 and earlier, published = 2 means published in 2012 and later); disease model is CKD or AKI (D); and method of model establishment is diabetes, cisplatin-induce AKI, DIO, or nephrectomy uremia (E). Figure S5. Forest plots for subgroup analyses of the CB1 antagonist and knockout on serum creatinine. Subgroup analyses were conducted stratified by the specie is rat or mouse (A); the intervention is antagonist or genetic (B); year of study published (C), (published = 1 means published in 2011 and earlier, published = 2 means published in 2012 and later); disease model is CKD or AKI (D); and method of model establishment is diabetes, cisplatin-induce AKI, DIO, or nephrectomy uremia (E). Figure S6. Forest plots for subgroup analyses of the CB1 antagonist and knockout on albuminuria. Subgroup analyses were conducted stratified by the specie is rat or mouse (A); the intervention is antagonist or genetic (B); year of study published (C), (published = 1 means published in 2011 and earlier, published = 2 means published in 2012 and later); disease model is CKD or AKI (D); and met [file 13098_2024_1283_MOESM1_ESM.zip › Supplemental Figure S5.jpg]

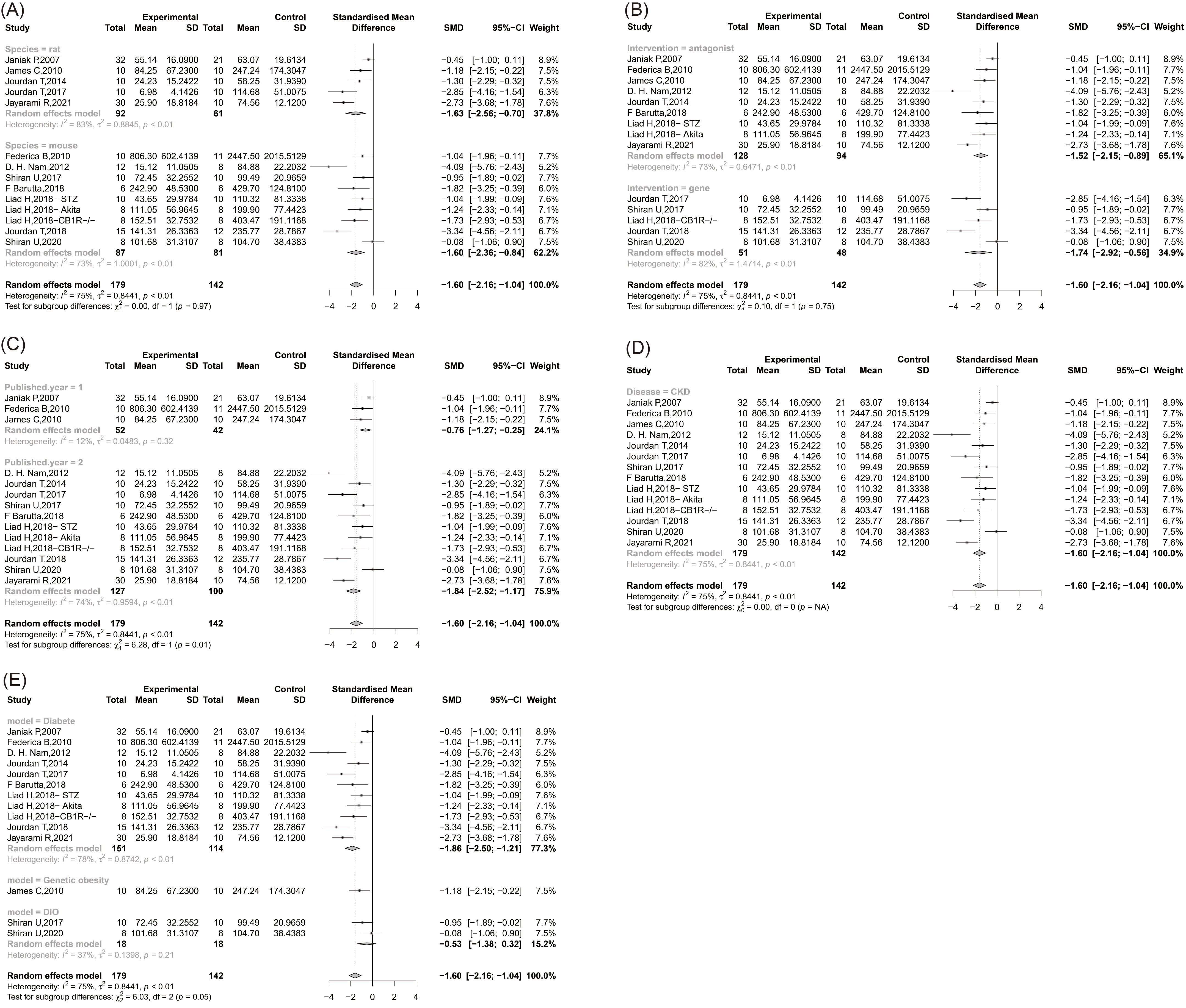

Supplement: Supplementary file 1 — Additional file 1: Figure S1. Quality assessment graph of the included studies: reviewers’ judgments about each risk of bias item for eligible studies based on SYRCLE’s RoB tool for animal studies. Figure S2. Forest plot for sensitivity analysis on CB1 antagonist and knockout primary outcomes including blood urea nitrogen (A), serum creatinine (B) and albuminuria (C). Figure S3. Forest plot for sensitivity analysis on CB2 agonist primary outcomes including blood urea nitrogen (A) and serum creatinine (B); CB2 antagonist and knockout primary outcomes including blood urea nitrogen (C); and serum creatinine (D). Figure S4. Forest plots for subgroup analyses of the CB1 antagonist and knockout on blood urea nitrogen. Subgroup analyses were conducted stratified by the specie is rat or mouse (A); the intervention is antagonist or genetic (B); year of study published (C), (published = 1 means published in 2011 and earlier, published = 2 means published in 2012 and later); disease model is CKD or AKI (D); and method of model establishment is diabetes, cisplatin-induce AKI, DIO, or nephrectomy uremia (E). Figure S5. Forest plots for subgroup analyses of the CB1 antagonist and knockout on serum creatinine. Subgroup analyses were conducted stratified by the specie is rat or mouse (A); the intervention is antagonist or genetic (B); year of study published (C), (published = 1 means published in 2011 and earlier, published = 2 means published in 2012 and later); disease model is CKD or AKI (D); and method of model establishment is diabetes, cisplatin-induce AKI, DIO, or nephrectomy uremia (E). Figure S6. Forest plots for subgroup analyses of the CB1 antagonist and knockout on albuminuria. Subgroup analyses were conducted stratified by the specie is rat or mouse (A); the intervention is antagonist or genetic (B); year of study published (C), (published = 1 means published in 2011 and earlier, published = 2 means published in 2012 and later); disease model is CKD or AKI (D); and met [file 13098_2024_1283_MOESM1_ESM.zip › Supplemental Figure S6.jpg]

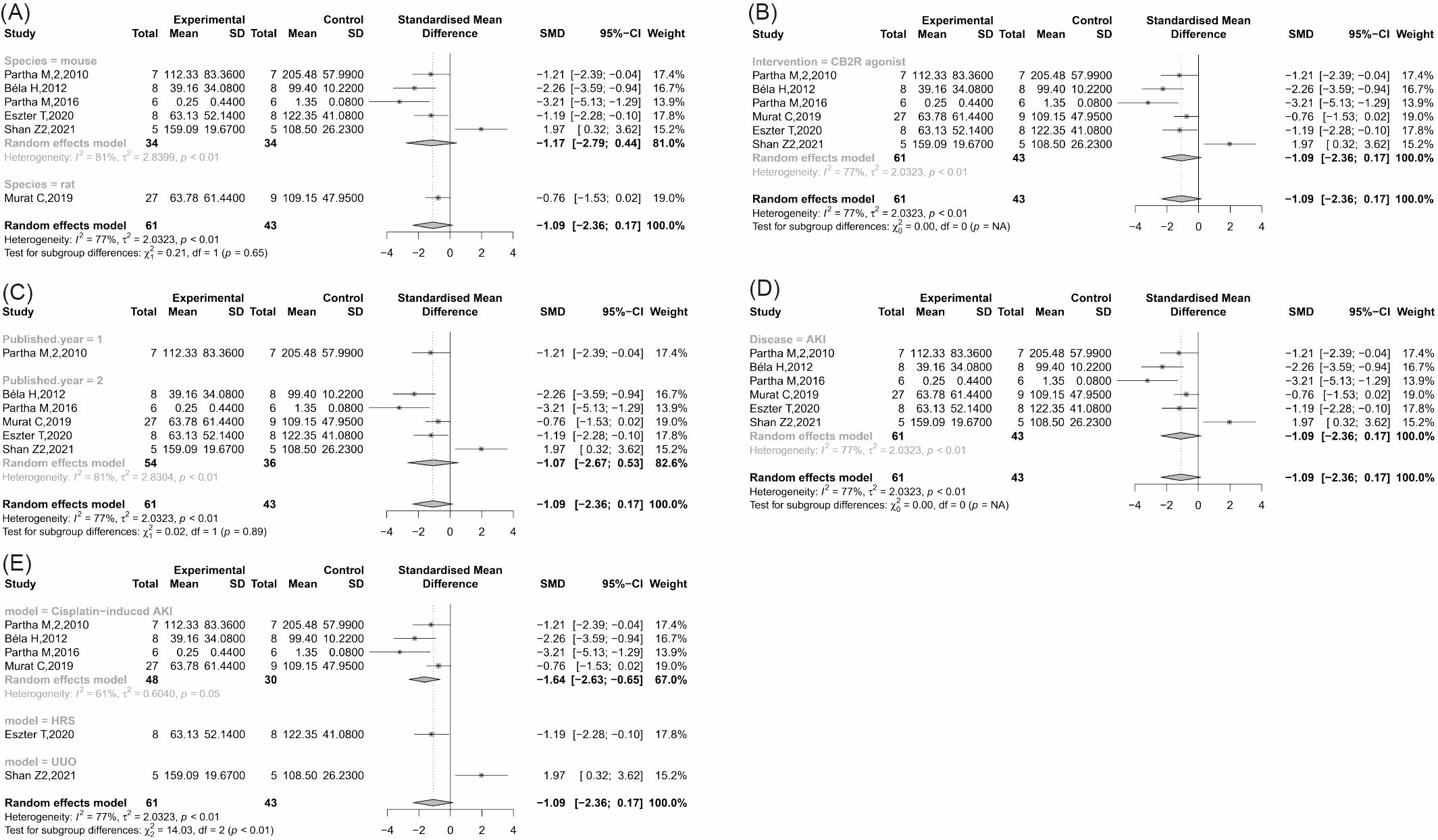

Supplement: Supplementary file 1 — Additional file 1: Figure S1. Quality assessment graph of the included studies: reviewers’ judgments about each risk of bias item for eligible studies based on SYRCLE’s RoB tool for animal studies. Figure S2. Forest plot for sensitivity analysis on CB1 antagonist and knockout primary outcomes including blood urea nitrogen (A), serum creatinine (B) and albuminuria (C). Figure S3. Forest plot for sensitivity analysis on CB2 agonist primary outcomes including blood urea nitrogen (A) and serum creatinine (B); CB2 antagonist and knockout primary outcomes including blood urea nitrogen (C); and serum creatinine (D). Figure S4. Forest plots for subgroup analyses of the CB1 antagonist and knockout on blood urea nitrogen. Subgroup analyses were conducted stratified by the specie is rat or mouse (A); the intervention is antagonist or genetic (B); year of study published (C), (published = 1 means published in 2011 and earlier, published = 2 means published in 2012 and later); disease model is CKD or AKI (D); and method of model establishment is diabetes, cisplatin-induce AKI, DIO, or nephrectomy uremia (E). Figure S5. Forest plots for subgroup analyses of the CB1 antagonist and knockout on serum creatinine. Subgroup analyses were conducted stratified by the specie is rat or mouse (A); the intervention is antagonist or genetic (B); year of study published (C), (published = 1 means published in 2011 and earlier, published = 2 means published in 2012 and later); disease model is CKD or AKI (D); and method of model establishment is diabetes, cisplatin-induce AKI, DIO, or nephrectomy uremia (E). Figure S6. Forest plots for subgroup analyses of the CB1 antagonist and knockout on albuminuria. Subgroup analyses were conducted stratified by the specie is rat or mouse (A); the intervention is antagonist or genetic (B); year of study published (C), (published = 1 means published in 2011 and earlier, published = 2 means published in 2012 and later); disease model is CKD or AKI (D); and met [file 13098_2024_1283_MOESM1_ESM.zip › Supplemental Figure S7.jpg]

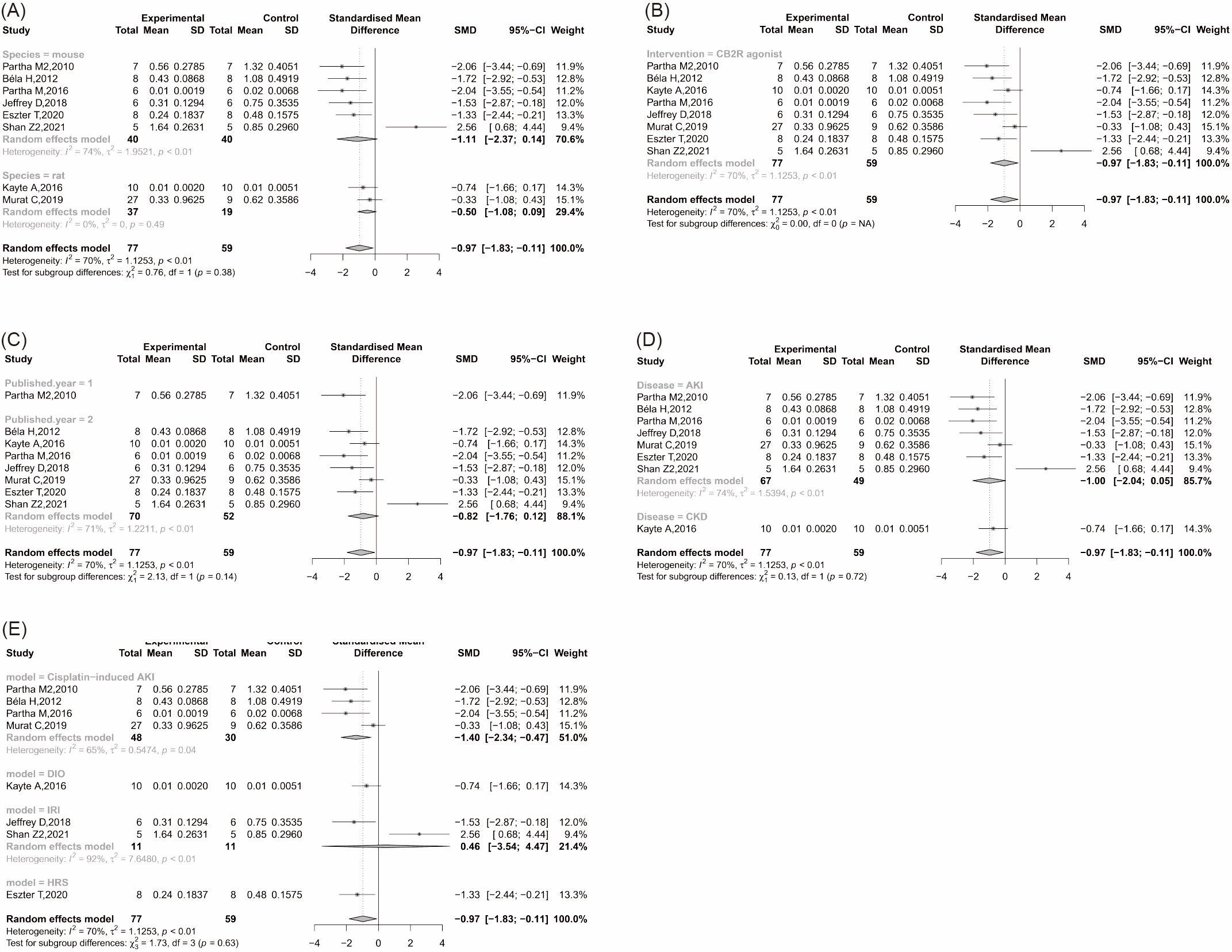

Supplement: Supplementary file 1 — Additional file 1: Figure S1. Quality assessment graph of the included studies: reviewers’ judgments about each risk of bias item for eligible studies based on SYRCLE’s RoB tool for animal studies. Figure S2. Forest plot for sensitivity analysis on CB1 antagonist and knockout primary outcomes including blood urea nitrogen (A), serum creatinine (B) and albuminuria (C). Figure S3. Forest plot for sensitivity analysis on CB2 agonist primary outcomes including blood urea nitrogen (A) and serum creatinine (B); CB2 antagonist and knockout primary outcomes including blood urea nitrogen (C); and serum creatinine (D). Figure S4. Forest plots for subgroup analyses of the CB1 antagonist and knockout on blood urea nitrogen. Subgroup analyses were conducted stratified by the specie is rat or mouse (A); the intervention is antagonist or genetic (B); year of study published (C), (published = 1 means published in 2011 and earlier, published = 2 means published in 2012 and later); disease model is CKD or AKI (D); and method of model establishment is diabetes, cisplatin-induce AKI, DIO, or nephrectomy uremia (E). Figure S5. Forest plots for subgroup analyses of the CB1 antagonist and knockout on serum creatinine. Subgroup analyses were conducted stratified by the specie is rat or mouse (A); the intervention is antagonist or genetic (B); year of study published (C), (published = 1 means published in 2011 and earlier, published = 2 means published in 2012 and later); disease model is CKD or AKI (D); and method of model establishment is diabetes, cisplatin-induce AKI, DIO, or nephrectomy uremia (E). Figure S6. Forest plots for subgroup analyses of the CB1 antagonist and knockout on albuminuria. Subgroup analyses were conducted stratified by the specie is rat or mouse (A); the intervention is antagonist or genetic (B); year of study published (C), (published = 1 means published in 2011 and earlier, published = 2 means published in 2012 and later); disease model is CKD or AKI (D); and met [file 13098_2024_1283_MOESM1_ESM.zip › Supplemental Figure S8.jpg]

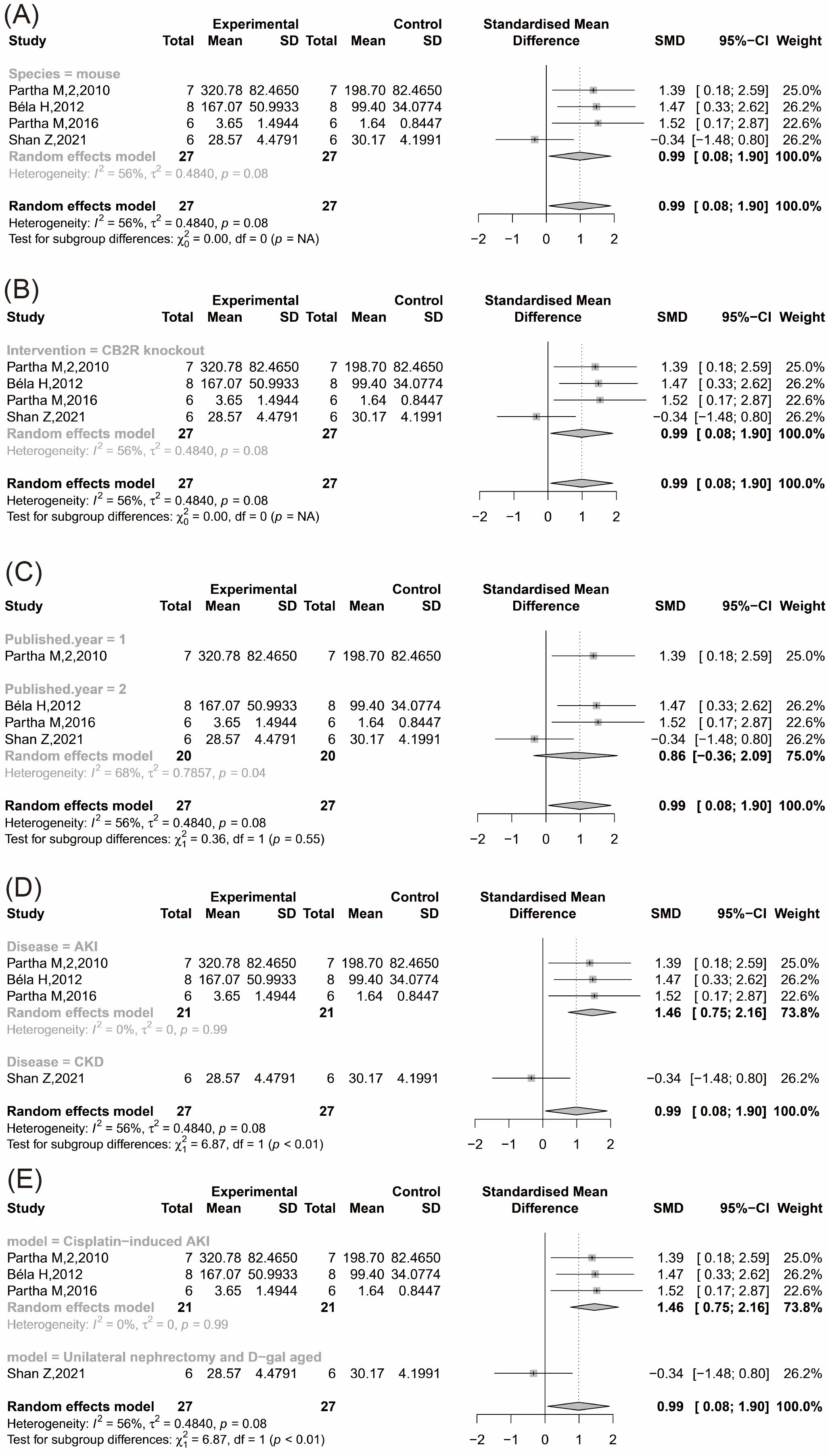

Supplement: Supplementary file 1 — Additional file 1: Figure S1. Quality assessment graph of the included studies: reviewers’ judgments about each risk of bias item for eligible studies based on SYRCLE’s RoB tool for animal studies. Figure S2. Forest plot for sensitivity analysis on CB1 antagonist and knockout primary outcomes including blood urea nitrogen (A), serum creatinine (B) and albuminuria (C). Figure S3. Forest plot for sensitivity analysis on CB2 agonist primary outcomes including blood urea nitrogen (A) and serum creatinine (B); CB2 antagonist and knockout primary outcomes including blood urea nitrogen (C); and serum creatinine (D). Figure S4. Forest plots for subgroup analyses of the CB1 antagonist and knockout on blood urea nitrogen. Subgroup analyses were conducted stratified by the specie is rat or mouse (A); the intervention is antagonist or genetic (B); year of study published (C), (published = 1 means published in 2011 and earlier, published = 2 means published in 2012 and later); disease model is CKD or AKI (D); and method of model establishment is diabetes, cisplatin-induce AKI, DIO, or nephrectomy uremia (E). Figure S5. Forest plots for subgroup analyses of the CB1 antagonist and knockout on serum creatinine. Subgroup analyses were conducted stratified by the specie is rat or mouse (A); the intervention is antagonist or genetic (B); year of study published (C), (published = 1 means published in 2011 and earlier, published = 2 means published in 2012 and later); disease model is CKD or AKI (D); and method of model establishment is diabetes, cisplatin-induce AKI, DIO, or nephrectomy uremia (E). Figure S6. Forest plots for subgroup analyses of the CB1 antagonist and knockout on albuminuria. Subgroup analyses were conducted stratified by the specie is rat or mouse (A); the intervention is antagonist or genetic (B); year of study published (C), (published = 1 means published in 2011 and earlier, published = 2 means published in 2012 and later); disease model is CKD or AKI (D); and met [file 13098_2024_1283_MOESM1_ESM.zip › Supplemental Figure S9.jpg]
